# Disruption of the crypt niche promotes outgrowth of mutated colorectal tumor stem cells

**DOI:** 10.1172/jci.insight.153793

**Published:** 2022-03-08

**Authors:** Stefan Klingler, Kuo-Shun Hsu, Guoqiang Hua, Maria Laura Martin, Mohammad Adileh, Timour Baslan, Zhigang Zhang, Philip B. Paty, Zvi Fuks, Anthony M.C. Brown, Richard Kolesnick

**Affiliations:** 1 Laboratory of Signal Transduction, Sloan Kettering Institute, Memorial Sloan Kettering Cancer Center, New York, New York, USA.; 2 Institute of Radiation Medicine, Fudan University, Shanghai, China.; 3 Cancer Biology and Genetics Program,; 4 Department of Surgery,; 5 Department of Surgery,; 6 Department of Radiation Oncology, and; 7 Department of Cell & Developmental Biology, Weill Cornell Medicine, New York, New York, USA.

**Keywords:** Oncology, Stem cells, Adult stem cells, Colorectal cancer, Tight junctions

## Abstract

Recent data establish a logarithmic expansion of leucine rich repeat containing G protein coupled receptor 5–positive (Lgr5^+^) colonic epithelial stem cells (CESCs) in human colorectal cancer (CRC). Complementary studies using the murine 2-stage azoxymethane–dextran sulfate sodium (AOM-DSS) colitis-associated tumor model indicate early acquisition of Wnt pathway mutations drives CESC expansion during adenoma progression. Here, subdivision of the AOM-DSS model into in vivo and in vitro stages revealed DSS induced physical separation of CESCs from stem cell niche cells and basal lamina, a source of Wnt signals, within hours, disabling the stem cell program. While AOM delivery in vivo under non-adenoma-forming conditions yielded phenotypically normal mucosa and organoids derived thereof, niche injury ex vivo by progressive DSS dose escalation facilitated outgrowth of Wnt-independent dysplastic organoids. These organoids contained 10-fold increased Lgr5^+^ CESCs with gain-of-function Wnt mutations orthologous to human CRC driver mutations. We posit CRC originates by niche injury–induced outgrowth of normally suppressed mutated stem cells, consistent with models of adaptive oncogenesis.

## Introduction

Despite significant improvement in prevention and therapy, colorectal cancer (CRC) remains one of the most common causes of cancer mortality worldwide (1). A better understanding of molecular mechanisms and impact of the environment driving onset of colorectal tumorigenesis is crucial to development of efficient preventive and therapeutic strategies. Recent studies describe intestinal stem cells (ISCs) as WNT-dependent cells localizing to the crypt base detectable by expression of the marker protein leucine rich repeat containing G protein coupled receptor 5 (Lgr5) (2). ISCs, intermingled in small intestinal crypts with Paneth cells, and in colon crypts with Paneth-like cKit^+^ cells, together with mesenchymal cells from the crypt stem cell niche (3, 4). This niche provides essential growth factors to the ISC. In the small intestine, Paneth cells provide Wnt, epidermal growth factor (EGF), and delta-like canonical notch ligands 1 and 4 (Dll1/Dll4) (5, 6). In contrast, colonic cKit^+^ cells provide EGF and Dll1/Dll4 but not Wnt proteins (5). Recently an alternative source for Wnt in the colon has been identified as Gli1-expressing mesenchymal cells that provide several Wnt ligands to the Lgr5^+^ colonic epithelial stem cell (CESC) (7). Current evidence paints a picture in which the ISC niche provides a complex of interacting morphogenic signals that function in ISC self-renewal and tissue homeostasis (8).

Inflammatory bowel disease (IBD) is characterized by chronic intestinal damage that compromises epithelial barrier function and leads to microbiota influx accompanied by inflammation (9). Patients with IBD manifest increased risk of developing colitis-associated cancer (CAC) of the colon (10), and the link between chronic inflammation and cancer, “the wound that never heals,” is well accepted (11, 12). Chronic inflammation is thought to induce multiple cancer-relevant events, including activation of growth factor signaling, genotoxic stress, reduced DNA repair, invasion, and metastasis (13). Many of these signaling events converge on activation of NF-κB, and STAT3, promoting premalignant cell proliferation and inhibition of apoptosis (13).

A well-established preclinical murine model of CAC involves treating mice with the extrinsic carcinogen azoxymethane (AOM) followed by administration of the sulfated polysaccharide dextran sulfate sodium (DSS) (14, 15). In this 2-stage model, DSS-induced chronic inflammation is thought to promote outgrowth of mutant cells to form adenomas and contributes to their progression toward malignancy (16). Genetic analysis shows that all AOM-DSS–induced adenomas carry activating mutations in the canonical Wnt signaling pathway (e.g., in β-catenin), consistent with evidence that aberrant Wnt signaling is a major driver of human CRC (17, 18). The role of inflammation in the AOM-DSS model has been studied in Toll-like receptor 4–knockout mice, which display significantly reduced colitis and severity of dysplasia (19). Genetic inactivation of a number of other proinflammatory factors, including IκB kinase, interleukin-6, and STAT3 (20–22), similarly shows reduced tumor incidence in the AOM-DSS model, but none prevent tumor formation entirely when inactivated. Mechanistically, in the mouse colon, DSS compromises colonic epithelial barrier function, allowing bacterial influx from the colon lumen and recruitment of immune cells, causing inflammation (15, 16). How DSS initiates this chain of events remains, for the most part, unclear.

Recent studies identify the Lgr5^+^ stem cell as a potential cell of origin for mouse adenoma development and human CRC (23, 24). In human CRC, the Lgr5^+^ stem cell population is expanded up to 10-fold (25, 26). To investigate the direct impact of DSS on CESCs and their progeny, we separated the 2 stages of the AOM-DSS model in time and space, treating mice with low-dose AOM under conditions in which tumor formation does not occur (14, 27), then applying DSS at a later time to organoids derived ex vivo from AOM-treated mice. DSS induces untransformed, wild-type CESCs in both organoid types to enter a stage of dormancy, which, if sustained, eventually leads to death of wild-type organoids. Concomitantly, in organoids from AOM-treated animals, DSS selects for outgrowth of a population of Wnt-autonomous, premalignant CESCs that rapidly dominate the organoid, restoring viability. This population displays gain-of-function mutations in the Wnt pathway orthologous to driver mutations found in human gastrointestinal (GI) malignancies and has multiple phenotypic properties of dysplasia and adenoma formation. These studies indicate that initial steps in colonic tumor formation can result from niche disruption in the absence of humoral immunity and inflammation.

## Results

### DSS induces rapid loss of CESC Lgr5.

**Figure 1:**
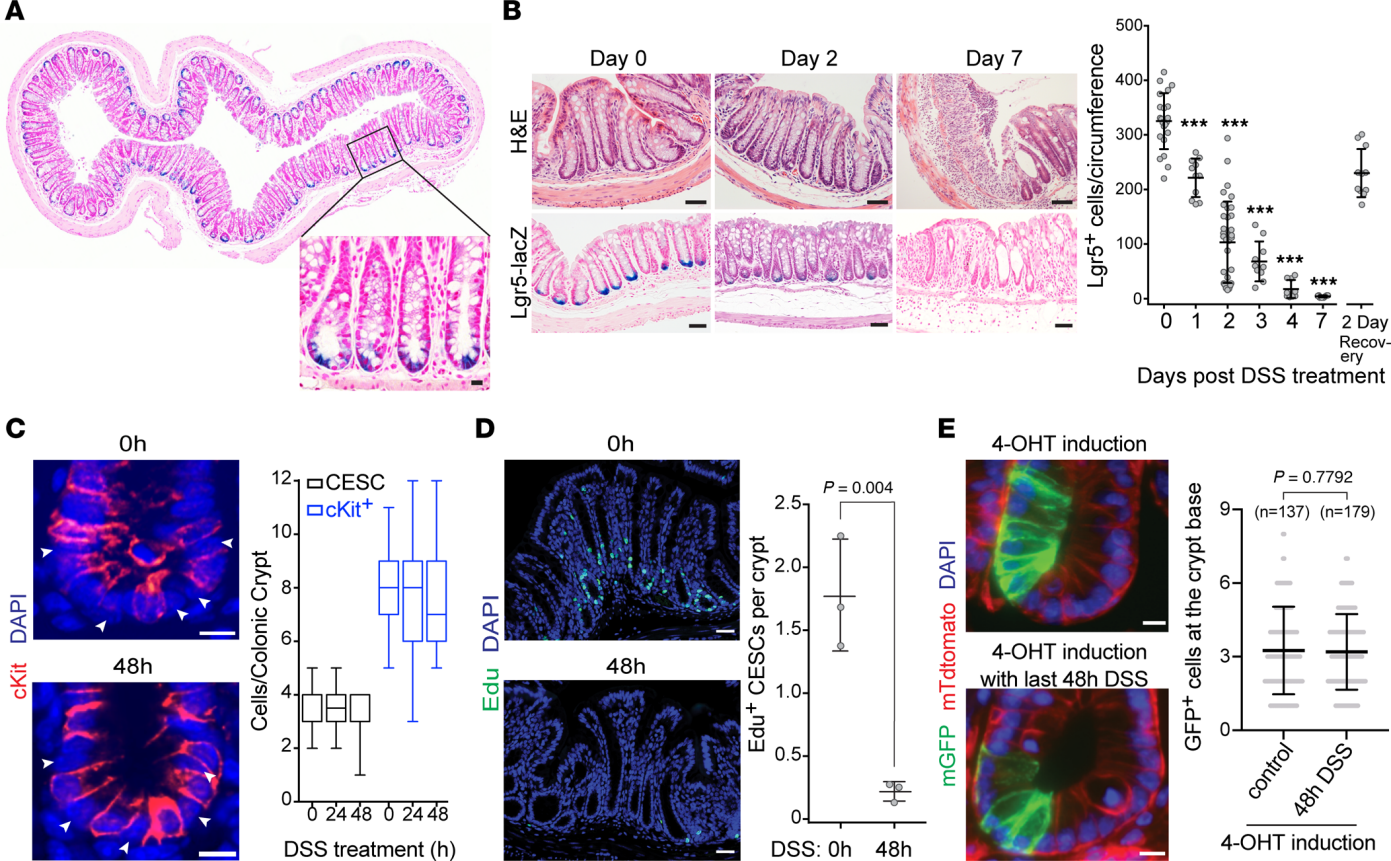
DSS induces loss of Lgr5 expression in CESCs. (**A**) Representative transverse section of distal colon from *Lgr5-lacZ* mice stained for lacZ. Scale bar = 10 μm. (**B**) Timing of loss of Lgr5^+^ stem cell *lacZ* signal after 1% DSS treatment. Left panel — H&E and lacZ staining of distal colons from *Lgr5-lacZ* mice at day 0, 2, and 7 after 1% DSS treatment, quantified in the right panel. Scale bar = 50 μm. For Lgr5 recovery, *Lgr5-lacZ* mice were treated with 1% DSS for 2 days followed by 2 days normal drinking water. Data (mean ± SEM) are from 6 mice/time point, evaluating 5 circumferences/mouse, except for *Lgr5-lacZ* recovery data, which are from 11 circumferences from 3 mice. ***P < 0.001 vs. day 0. Note, *P* < 0.001, 2-day recovery vs. day 2 DSS. (**C**) CESCs remain physically present at the crypt base after 2 days of 1% DSS treatment. White arrows identify CESCs between red fluorescent cKit^+^ cells in colonic crypts. Data (mean ± SD) are collated from 100 crypts (4 mice/time point), *P* > 0.05, 2-tailed Student’s *t* test. Scale bar = 10 μm. (**D**) Number of proliferating CESCs dramatically decreases at day 2 of 1% DSS treatment. S-phase cells were pulse-labeled with EdU for 2 hours before sacrifice. Scale bar = 50 μm. Data (mean ± SD) show representative results from 3 independent experiments. (**E**) Lineage tracing using *Lgr5-mTmG* transgenic mice demonstrates GFP^+^ CESCs at the crypt base after 2 days of 1% DSS treatment. Data (mean ± SD) are collated from 137 and 179 intact crypts for control and DSS groups, respectively, from 3 mice/group. *P* value, 2-tailed Student’s *t* test. Scale bar = 10 μm. The box plots depict the minimum and maximum values (whiskers), the upper and lower quartiles, and the median. The length of the box represents the interquartile range. 4-OHT, 4-hydroxytamoxifen.

We first examined the impact of DSS alone on Lgr5^+^ CESCs in vivo using Lgr5 reporter mice. We added 1% DSS to the drinking water of the *Lgr5-lacZ* knockin strain (2), and colonic tissue was processed for histology and lacZ detection after 2 and 7 days (Figure 1A). As in parental C57BL/6 mice, acute colitis was observed by day 7, evidenced by extensive loss of colonic crypts, ulceration, and mucosal macrophage infiltration (Figure 1B). At day 2, when neither colonic mucosa histology nor crypt number per circumference (Supplemental Figure 1A; supplemental material available online with this article; https://doi.org/10.1172/jci.insight.153793DS1) was detectably altered, dramatic reduction in lacZ-expressing CESCs was observed at the crypt base (Figure 1B and Supplemental Figure 1A). Untreated mice displayed 325 ± 11 lacZ^+^ CESCs per large intestinal circumference, which were reduced to 103 ± 13 CESCs (*P* < 0.001, mean ± SEM) by DSS treatment. Reduction in lacZ^+^ CESCs was progressive, reaching as few as 4 ± 1 CESCs/circumference at day 7. Loss of lacZ staining was reversible, however, as lacZ^+^ CESCs rapidly reappeared upon withdrawal of DSS from drinking water, attaining 71% of the normal level by day 2 of recovery (Figure 1B, right panel). Rapid DSS-induced reduction of CESC Lgr5 expression was confirmed in *Lgr5-EGFP-ires-CreERT2* reporter mice (Supplemental Figure 1B, day 2,4), which display stochastic GFP marker expression in murine CESCs and are therefore suboptimal for precise CESC quantitation (28, 29). In situ hybridization (ISH) for *Lgr5* mRNA showed a rapid decrease in *Lgr5* mRNA induced by DSS in concordance with loss of lacZ staining (Supplemental Figure 1C). In contrast, no difference in cKit^+^ niche cell number after DSS treatment was detected by cKit immunofluorescence (0 hours: 8.1 ± 1.4; 24 hours: 7.8 ± 2.0; 48 hours: 7.6 ± 1.6 cKit^+^ cells/crypt after DSS; Figure 1C). As Lgr5^+^ crypt base columnar cells of the small intestine are prone to apoptosis (2, 30), we initially tested whether DSS led to CESC deletion by apoptosis. This was measured by immunohistochemistry for the active fragment of caspase-3, a commonly used assay for evaluating apoptotic induction in the GI mucosa (30). As no apoptosis was visible at 0 hours, 24 hours, or 48 hours after DSS treatment, we conclude that DSS-induced loss of Lgr5 positivity was not mediated by CESC apoptosis (Supplemental Figure 2A). Alternatively, CESCs might be exhausted by enhanced stimulation of terminal differentiation. However, immunohistochemistry for differentiation markers — mucin2 (for goblet cells), carbonic anhydrase IV (for enterocytes), and chromogranin A (for enteroendocrine cells) — failed to reveal enhanced DSS-induced CESC differentiation (Supplemental Figure 2B). While these data indicate that neither apoptotic death nor terminal differentiation caused the apparent loss of murine Lgr5^+^ cells, the stem cell compartment nonetheless showed an 88% ± 2% decrease in proliferation after 1% DSS treatment, as measured by EdU incorporation assay (Figure 1D). Closer inspection of the base of the crypt revealed that despite loss of Lgr5 expression, DAPI^+^ CESCs were still clearly visible interspersed between cKit^+^ cells, and the number of DAPI^+^ cells residing between cKit^+^ cells was preserved (Figure 1C; 0 hours: 3 1; 24 hours: 4 ± 1; 48 hours: 3 ± 1 DAPI^+^ cells/crypt after DSS). This suggests that physical loss of CESCs had not occurred upon DSS treatment.

To confirm this observation, we performed lineage tracing using *Lgr5-ires-CreERT2(3′UTR)/Rosa26-mTmG* mice in which the *ires*-*CreERT2* cassette is inserted into the 3′UTR of the *Lgr5* locus (31). With an mT/mG dual-color reporter driven by a *CMV* *β-actin* enhancer promoter (*pCA*), these animals display constitutive red fluorescence (membrane-targeted tdTomato, mT) prior to tamoxifen induction, which results in a Cre-mediated excision of the *loxP-mT-loxP* sequence, allowing the *pCA* promoter to drive expression of membrane-targeted enhanced GFP (mG) initiated in CESCs by the *Lgr5* promoter (32). Conversion of the red to a green signal was observed in CESCs at the crypt base (positions +1–+4) of wild-type mice upon Cre induction at day 4 and extended into the transit cell–containing region at day 6 (Figure 1E, left panel), with 3.3 ± 0.2 GFP^+^ CESCs on average observed at the crypt base (Figure 1E, right panel). Treatment with DSS beginning at day 4 after Cre induction did not affect the number of GFP^+^ CESCs (3.2 ± 0.1 cells) detected at day 6 postinduction. While these data show DSS induced clinically relevant features of colitis within 5 to 7 days, as published (33), within the first 48 hours of DSS treatment, preceding inflammation, rapid but reversible loss of well-established “stemness” properties of CESCs (Lgr5^+^ expression, proliferation) occurred without detectable loss of cell number. This suggests that CESCs enter a state of “dormancy” in response to DSS, preceding pathologic changes of colitis.

### DSS treatment rapidly disrupts the stem cell niche in vivo.

**Figure 2:**
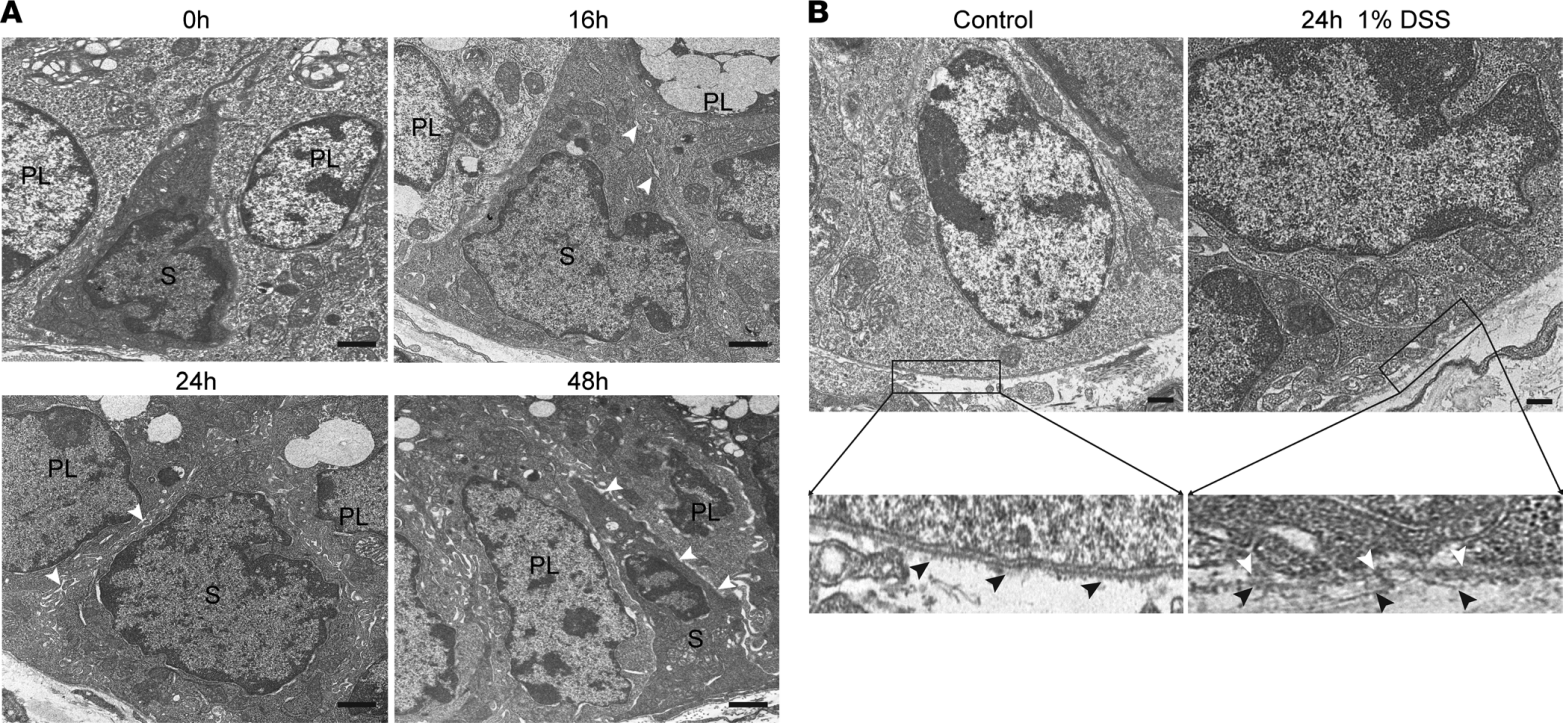
DSS treatment disrupts the stem cell niche. (**A**) Effect of DSS on cell-cell junctional complexes in colonic epithelium at the crypt base. *Lgr5-lacZ* mice were treated with 1% DSS, and distal colonic sections were subjected to TEM. Scale bar = 500 nm. White arrows indicate typical regions of separation of CESCs from Paneth-like cells. PL, Paneth-like cells, which are identified by presence of granules; S, stem cell. (**B**) *Lgr5-lacZ* mice were treated with 1% DSS for 24 hours, and distal colonic sections were subjected to TEM. Black arrows indicate basal lamina at the crypt base. White arrows indicate regions of separation of CESCs from basal lamina. Scale bar = 500 nm.

Critical cell-cell contacts exist between Lgr5^+^ stem cells and cognate cKit^+^ niche cells, which provide factors, including EGF and the Notch ligands Dll1 and Dll4, to maintain CESC “stemness” (3, 5). Based on a report of rapid DSS impact on tight junctions (33), we used high-magnification transmission electronic microscopy (TEM) to examine rapid alterations in cell-cell contacts in the CESC niche. Beginning at 16 hours after DSS treatment, an increase in the paracellular space between the Lgr5^+^ CESCs and its cKit^+^ niche cell was detected, and over 24–48 hours of DSS exposure, there was progressive widening of the paracellular space leading to almost complete separation of the 2 cell types (Figure 2A and Supplemental Figure 3). Furthermore, separation of Lgr5^+^ CESCs from their neighboring niche cells was not restricted to the crypt. Following initial separation of CESCs from cKit^+^ niche cells, beginning at 24 hours, CESCs became disconnected from the basal lamina, a repository for Wnt3a protein presentation to CESCs (Figure 2B). These events are consistent with the reported loss of epithelial barrier function in response to DSS (33) well before visible infiltration of immune cells (Figure 1B). Moreover, onset of niche cell separation occurred before significant loss of Lgr5 protein or mRNA expression was observed (data not shown), raising the possibility that loss of Lgr5 expression might result from the loss of niche integrity.

To confirm an effect of physical niche disruption on Wnt signaling, we performed β-catenin immunostaining of mouse colonic specimens with or without 2 days of DSS treatment, evaluating β-catenin nuclear translocation, a key event mediating transcription of Wnt target genes (34). In control untreated mice, we found nuclear β-catenin resided specifically in the proliferative compartment of colonic crypts, with on average 1.3 ± 0.6 nuclear β-catenin–containing cells per crypt, which are data similar to those reported by Clevers and coworkers (35). In colonic crypts of DSS-treated mice, however, we observed >10-fold reduction in number of nuclear β-catenin^+^ cells at the crypt base, indicating DSS-induced niche disruption inactivates Wnt signaling (Supplemental Figure 4). Additionally, we showed dramatic reduction in expression of well-known Wnt target genes, including Ascl2, Axin2, and Lgr5, in DSS-treated wild-type organoids, indicating multiple critical stemness genes of the Wnt program are concomitantly inactivated by DSS treatment (36) (Supplemental Figure 5).

### DSS reduces growth and survival of normal colon-derived organoids but not of those from adenomas.

**Figure 3:**
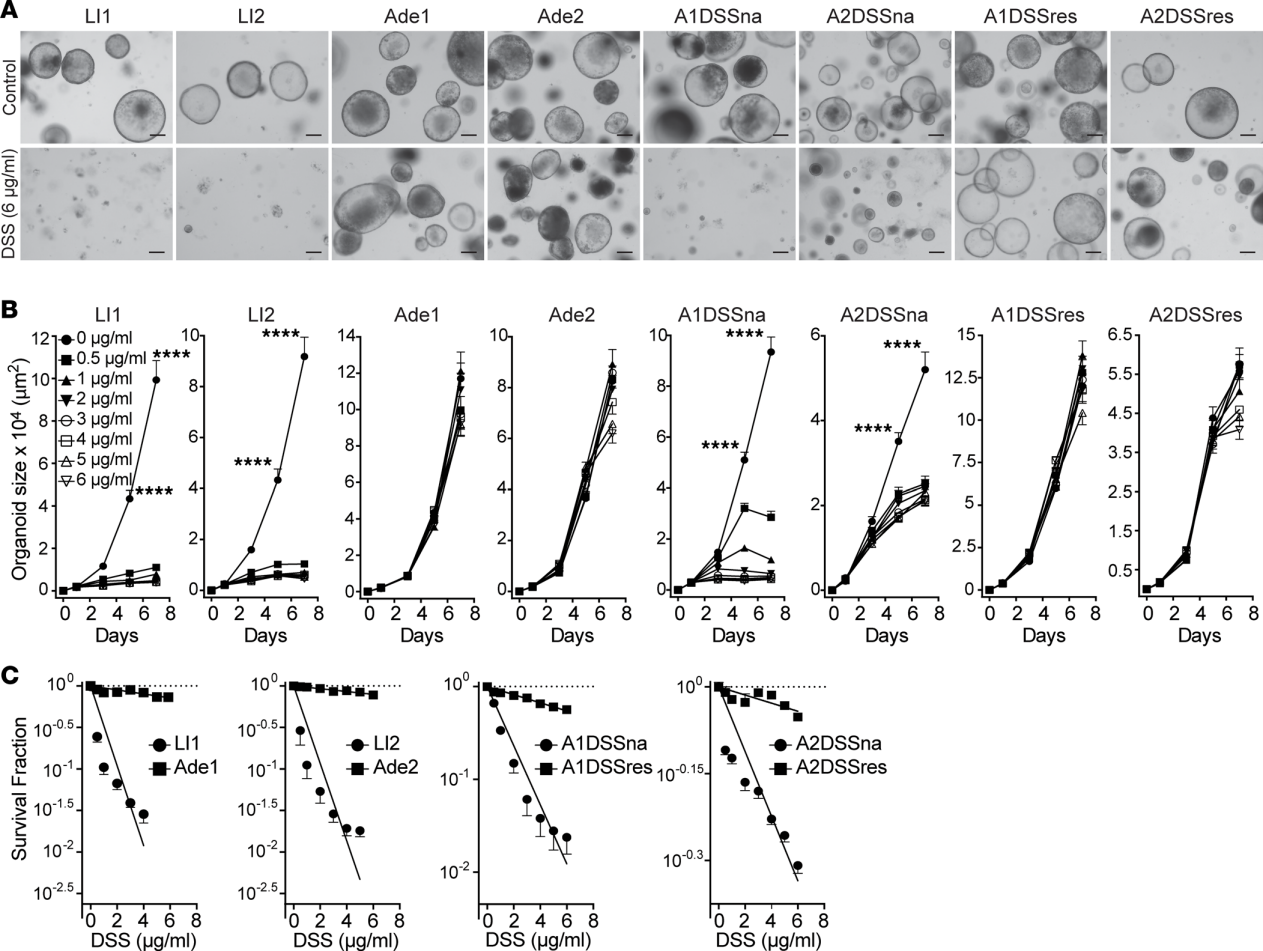
In vitro DSS treatment selects for DSS-resistant subclones. (**A**) Representative images of LI1, LI2, A1DSSna, A2DSSna, A1DSSres, A2DSSres, Ade1, and Ade2 organoids either left untreated (control) or treated with 6 μg/mL DSS for 7 days. Scale bar = 200 μm. (**B**) DSS dose-response growth curves from organoids treated with 0–6 μg/mL DSS for 7 days. Data (mean ± SEM) were acquired on day 1, 3, 5, and 7, *P* < 0.0001 (****), 1-way ANOVA. (**C**) Transformation of the 7 day data (mean ± SEM) in **B** as per the SHMT algorithm.

To test direct effects of DSS on colonic epithelial cells in vitro, we established colonic organoid cultures from crypt-like tissue fragments from 2 healthy, untreated mice (LI1 and LI2) and from 2 colonic adenomas (Ade1 and Ade2) derived from mice exposed in vivo to the AOM-DSS regimen, as described (37, 38). As with the majority of AOM-DSS–induced adenomas (39), both lines were found to contain gain-of-function mutations in β-catenin (G34E and S37F, respectively), yielding constitutively active Wnt signaling. For these experiments, organoids were mechanically disrupted into crypt-like fragments, then allowed to recover overnight, and DSS was added to standard organoid growth media at a range of concentrations from 0 to 6 μg/mL. While DSS severely inhibited growth of wild-type LI1 and LI2 organoids, even at the lowest concentration tested (0.5 μg/mL) (Figure 3, A and B), Ade1 and Ade2 adenoma organoid lines were completely DSS resistant, even at the highest dose of 6 μg DSS/mL (Figure 3, A and B).

### DSS selection of organoids from AOM-treated mice confers adenoma-like features.

The C57BL/6 mouse strain used for these studies does not develop adenomas from treatment with AOM as single agent, even with multiple injections, unless there is subsequent DSS challenge (27, 40). To investigate the effects of AOM, C57BL/6 mice were injected intraperitoneally with 10 mg/kg AOM weekly for 5 consecutive weeks (41). At 9–11 weeks after the last AOM injection, colonic tissue was processed for pathologic analysis, and colonic crypts were harvested for organoid development.

Consistent with published data, detailed analysis by a mouse pathologist, board certified by the American College of Veterinary Pathologists, revealed phenotypically normal colonic tissue, with no precancerous lesions and a lack of aberrant crypt foci, the earliest identifiable lesion (42) (Supplemental Figure 6). In agreement with this lack of pathology, organoids derived from 2 AOM-treated C57BL/6 mice (designated A1DSS naive, A1DSSna; and A2DSS naive, A2DSSna) and cultured in 50% Wnt3a-conditioned medium (v/v), 10% R-spondin1–conditioned medium, 5% Noggin-conditioned medium, and recombinant EGF (hereafter termed WENR, as published by Clevers and colleagues, refs. 37, 38, 43) were phenotypically indistinguishable from untreated large intestinal organoids in terms of morphology, growth rate, conditions for subculturing, and maintenance requirements (data not shown) (38).

To address whether these phenotypically similar organoids respond differently from wild-type organoids to DSS challenge, growth and survival of L1 and L2 were compared with A1DSSna and A2DSSna. Initial studies directly compared effects of increasing doses of DSS (0.5–6 μg/mL) on the size of actively growing organoids. While wild-type LI1 and LI2 organoids were sensitive to relatively low doses of DSS (0.5 μg/mL DSS) (Figure 3, A and B), A1DSSna and A2DSSna organoids displayed minimal to moderate DSS resistance. However, upon exposure incrementally over several months to escalating DSS doses up to 2.5 μg/mL, a strategy that prevented growth of wild-type organoids, DSS-resistant subclones of the AOM-derived lines were obtained, as detailed in Methods. These were termed A1DSS resistant (A1DSSres) and A2DSS resistant (A2DSSres). The resistant subclones were capable of continuous expansion even in the highest DSS concentration tested (6 μg/mL), a feature shared with Ade1 and Ade2 adenoma-derived organoids (Figure 3, A and B).

To provide a quantitative measure of DSS resistance, organoids were treated as above, and survival was calculated by determining the number of living organoids on day 7 after DSS. Numbers of surviving organoids were transformed by nonlinear regression fitting by a modification of the single hit multitarget (SHMT) algorithm, yielding a single “D_0_-like” value that serves as a numerical estimate of DSS sensitivity. The SHMT algorithm has been used extensively to quantify inherent resistance of cell populations and tissues to ionizing radiation (44–46) and was adapted here to compare inherent DSS resistance. Note, the higher the D_0_ value, the greater the DSS resistance (47). Figure 3C shows that LI1, LI2, A1DSSna, and A2DSSna organoids were highly sensitive to DSS (μg/mL) with D_0_ values (μg/mL) of LI1 = 0.9; LI2 = 0.9; A1DSSna = 1.4; and A2DSSna = 7.8; whereas DSS-resistant A1DSSres, A2DSSres, Ade1, and Ade2 organoids were able to withstand DSS with D_0_ values (μg/mL) of A1DSSres = 9.9; A2DSSres = 62.0; Ade1 = 18.1; and Ade 2 = 24.8. These data thus depict a logarithmic increase in DSS tolerance in A1DSSres and A2DSSres organoids after DSS desensitization compared with wild-type LI organoids. Taken together, these investigations show that large intestinal organoids from untreated wild-type or DSS-naive AOM-treated mice are DSS sensitive, displaying decreased growth and reduced survival in its presence. However, DSS-desensitized organoids from AOM-treated mice are capable of proliferation in high DSS conditions and are refractory to DSS-induced death, as is also the case for adenoma organoids.

**Figure 4:**
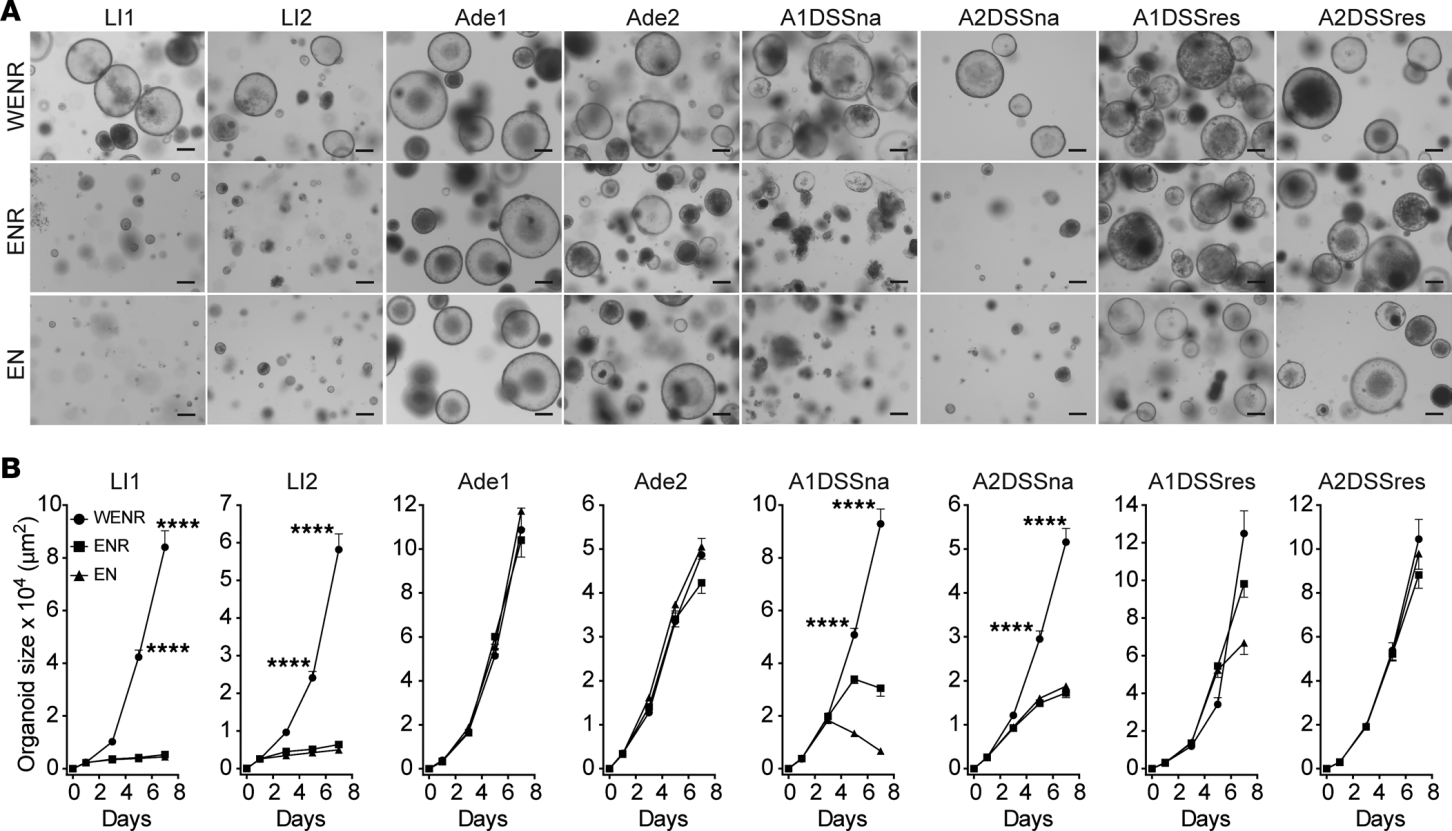
In vitro DSS treatment selects Wnt signaling–autonomous subclones. (**A**) Representative images of LI1, LI2, A1DSSna, A2DSSna, A1DSSres, A2DSSres, Ade1, and Ade2 organoids treated with WENR, ENR, or EN growth media for 7 days. Scale bar = 200 μm. (**B**) Growth curve data (mean ± SEM) from organoids treated with WENR, ENR, or EN. *P* < 0.0001 (****), 1-way ANOVA.

While exogenous Wnt is obligatory for proliferation and suppression of differentiation of wild-type ISCs and for large intestinal organoid growth, Wnt signaling autonomy is a hallmark of human colon adenomas and CRCs and of organoid models derived thereof (37, 38, 48). We therefore examined whether in vivo AOM administration followed by ex vivo DSS desensitization influenced A1DSSres/A2DSSres dependency on exogenous Wnt and R-spondin1 ligands. While large intestinal organoids require WENR for growth in vitro (3, 4, 38, 48–50), adenoma organoids require only addition of EGF and Noggin (EN) to basic media for their growth in culture (48, 51). For these investigations, organoid lines were mechanically disrupted, plated in Matrigel, allowed to recover overnight, and then treated with growth media supplemented with WENR; EGF, Noggin, and R-spondin1 (ENR); or EN. LI1 and LI2 wild-type organoids required Wnt, as they were incapable of growth in ENR or EN, consistent with previous publications (3, 48). A1DSSna and A2DSSna showed short-term survival and growth in ENR- or EN-supplemented media but, like LI1 and LI2, became rapidly growth arrested (Figure 4, A and B). In contrast, DSS-resistant A1DSSres and A2DSSres lines showed largely unperturbed growth in ENR and EN media, a feature shared with adenoma organoids (Figure 4, A and B), indicating autonomy from exogenous addition of the stem cell growth factors Wnt and R-spondin1. These data indicate that Wnt independence of the organoids is acquired concomitantly with DSS resistance.

Consistent with the above data, wild-type large intestinal organoids display morphologic features distinct from adenoma organoids. After 5 days in growth media, H&E sections showed that LI1 and LI2 grew primarily as a single epithelial monolayer with a clearly visible lumen with LI1 and LI2 displaying 1.17 ± 0.02 and 1.20 ± 0.02 (mean ± SEM) cell layers, respectively (Supplemental Figure 7, A and B). Similarly, A1DSSna and A2DSSna grew predominantly as a monolayer composed of 1.25 ± 0.02 and 1.23 ± 0.01 cell layers. In contrast, A1DSSres and A2DSSres showed a multi–cell layer phenotype with 1.78 ± 0.04 and 1.78 ± 0.04 cell layers, respectively. This is comparable to the adenoma lines Ade1 and Ade2 that manifested 1.79 ± 0.04 and 1.75 ± 0.03 cell layers, respectively (Supplemental Figure 7, A and B). Thus, selection for DSS resistance confers a dysplastic multicellular phenotype on organoids derived from AOM-treated mice that otherwise appear phenotypically and morphologically normal.

Another feature of benign and malignant tumors replicated in our organoid culture is an increased nuclear-to-cytoplasm (NC) ratio (52, 53). Wild-type L1 and L2 organoids displayed an NC ratio of 0.57 ± 0.01 and 0.55 ± 0.01 (mean ± SEM), respectively (Supplemental Figure 7C). Similarly, A1DSSna and A2DSSna organoids showed an NC ratio of 0.58 ± 0.01 and 0.62 ± 0.01. In contrast, A1DSSres and A2DSSres manifested increased NC ratios of 0.88 ± 0.01 and 0.88 ± 0.01, respectively, comparable to those of Ade1 and Ade2 adenoma organoids, 0.87 ± 0.01 and 0.86 ± 0.01, respectively (*P* < 0.0001 vs. wild-type and naive organoids each) (Supplemental Figure 7C). These studies indicate that organoids from colorectal tissue exposed in vivo to AOM are indistinguishable from organoids derived from untreated wild-type tissue, and upon DSS selection they transition to a frankly dysplastic/tumorigenic like phenotype.

### DSS selection expands Wnt-autonomous Lgr5^+^ stem cells.

**Figure 5:**
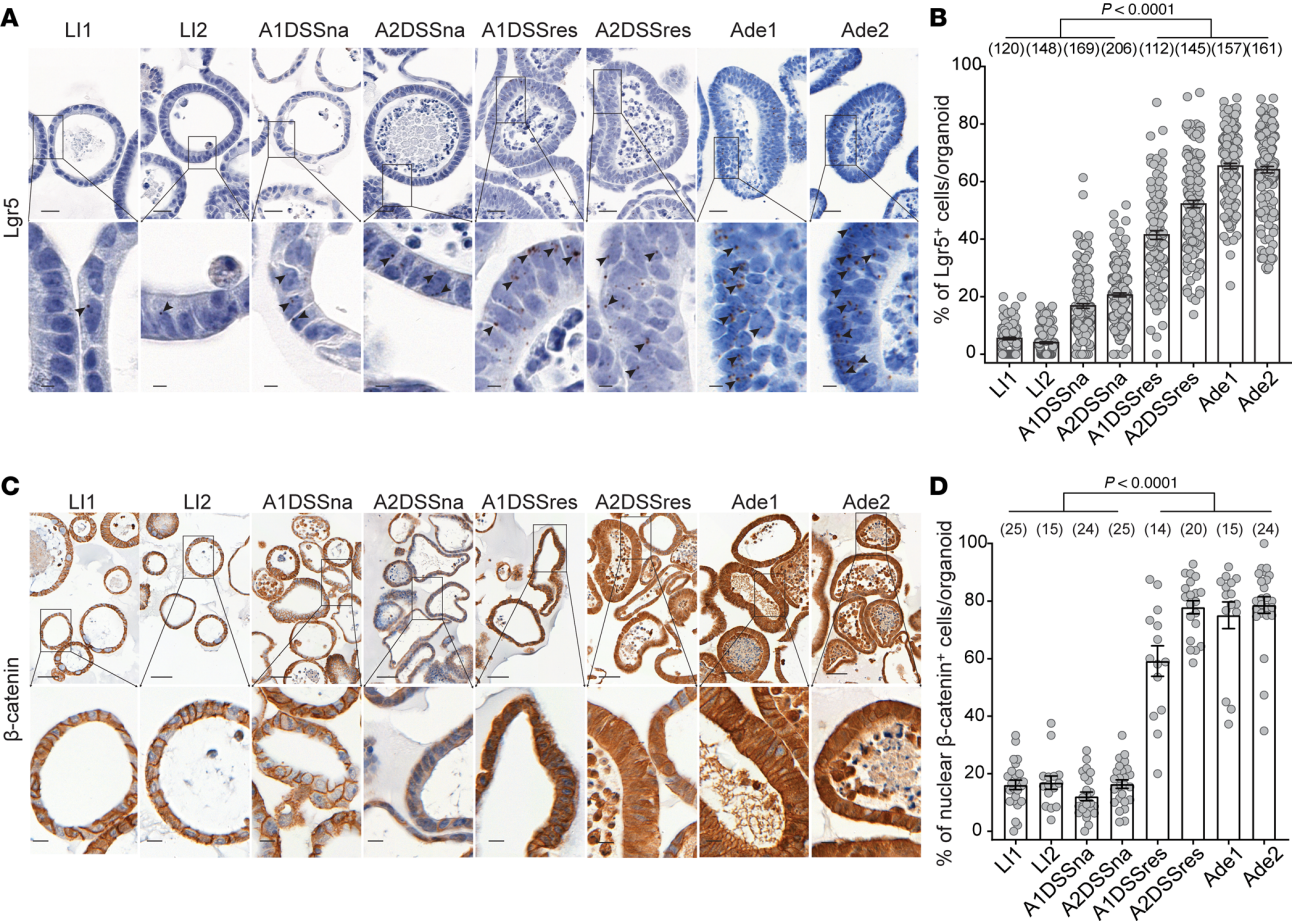
DSS-induced niche disruption leads to an expanded Lgr5^+^ cell population and increased Wnt signaling autonomy. (**A**) Representative Lgr5 ISH images in LI1, LI2, A1DSSna, A2DSSna, A1DSSres, A2DSSres, Ade1, and Ade2 organoids. Arrowheads point to *Lgr5* mRNA punctae within cells. Scale bar = 20 μm; scale bar in magnification = 5 μm. (**B**) Quantification of Lgr5^+^ cells per organoid (**A**). Each data point represents an individual organoid. Data (mean ± 95% CI) are collated from total number of organoids analyzed, as represented in parentheses for each line. (**C**) Representative IHC images for β-catenin in LI1, LI2, A1DSSna, A2DSSna, A1DSSres, A2DSSres, Ade1, and Ade2 organoids. Scale bar = 50 μm; scale bar in magnification = 10 μm. (**D**) Quantification of nuclear β-catenin per organoid (**C**). Each data point represents an individual organoid. Data (mean ± SD) are collated from total number of organoids analyzed, as represented in parentheses for each line. One-way ANOVA was used to obtain the *P* values.

Recent studies have found that the Lgr5^+^ stem cell population is logarithmically expanded in murine and human adenomas and carcinomas (25) and correlates with poor CRC prognosis (26). Accurate quantitation of the Lgr5^+^ population in colonic organoids is subject to limitations because in vitro culture conditions require addition of exogenous Wnt to sustain survival, artificially increasing the percentage of Lgr5^+^ cells compared with Lgr5^+^ CESC numbers in vivo (38). To ensure a physiologic complement of Lgr5^+^ cells in wild-type organoids, it was necessary to remove exogenous Wnt from media for 16 hours (data not shown). Under these conditions, the Lgr5^+^ cell population in LI1 and LI2 represented 5.5% ± 0.4% (mean ± SEM) and 4.0% ± 0.4% of total cells, respectively, a proportion comparable to that in crypts in vivo (25) (Figure 5, A and B). In contrast, in the absence of exogenous Wnt, Ade1 and Ade2 contained 65.4% ± 1.0% and 64.1% ± 1.1% Lgr5^+^ cells, respectively, similar to the logarithmic increase seen in human adenomas (25). Although pathologic analysis reported A1DSSna and A2DSSna as morphologically normal, these organoids nevertheless displayed baseline elevation of Lgr5^+^ cells of 16.8% ± 0.8% and 20.6% ± 0.7% of the total population, respectively, with a further increase by induction of DSS resistance in A1DSSres to 41.5% ± 1.5% and in A2DSSres to 52.2% ± 1.3% (*P* < 0.0001 each vs. naive) (Figure 5, A and B).

Since Lgr5 is both a stem cell marker and a Wnt target gene, the increase in Lgr5^+^ cell content suggested augmented autonomous activation of the canonical Wnt program. To investigate ligand-independent Wnt activation in A1DSSres and A2DSSres, we examined β-catenin expression in organoids starved of exogenous Wnt3a ligand, as described above. We found β-catenin predominantly localizes to cell membranes with very weak or absent cytoplasmic and nuclear signals in LI1, LI2, A1DSSna, and A2DSSna organoids with no statistical difference between these lines (A1DSSna: 12% ± 7%; A2DSSna: 16% ± 7%; LI1: 16% ± 8%; LI2: 17% ± 9%; mean ± SD). In contrast, in A1DSSres and A2DSSres organoids, β-catenin is enriched in cytoplasm in addition to cell membranes, with low to medium nuclear expression, a pattern similar to that in human hyperplastic polyps or adenomas with low-grade dysplasia (54–59). Cytoplasmic/nuclear enrichment frequencies in A1DSSres and A2DSSres were comparable to the elevated levels in Ade1 and Ade2 (A1DSSres: 59% ± 20%; A2DSSres: 78% ± 10%; Ade1: 75% ± 18%; Ade2: 79% ± 14%; Figure 5, C and D).

Taken together, these data suggest that AOM administration alters CESCs on a molecular level, nudging them along the path toward tumor transformation, changes in of themselves insufficient for induction of histologic alteration (Supplemental Figure 6) absent a second, tumor-promoting stimulus, provided ex vivo by DSS selection.

### DSS selects for CRC driver mutations.

**Figure 6:**
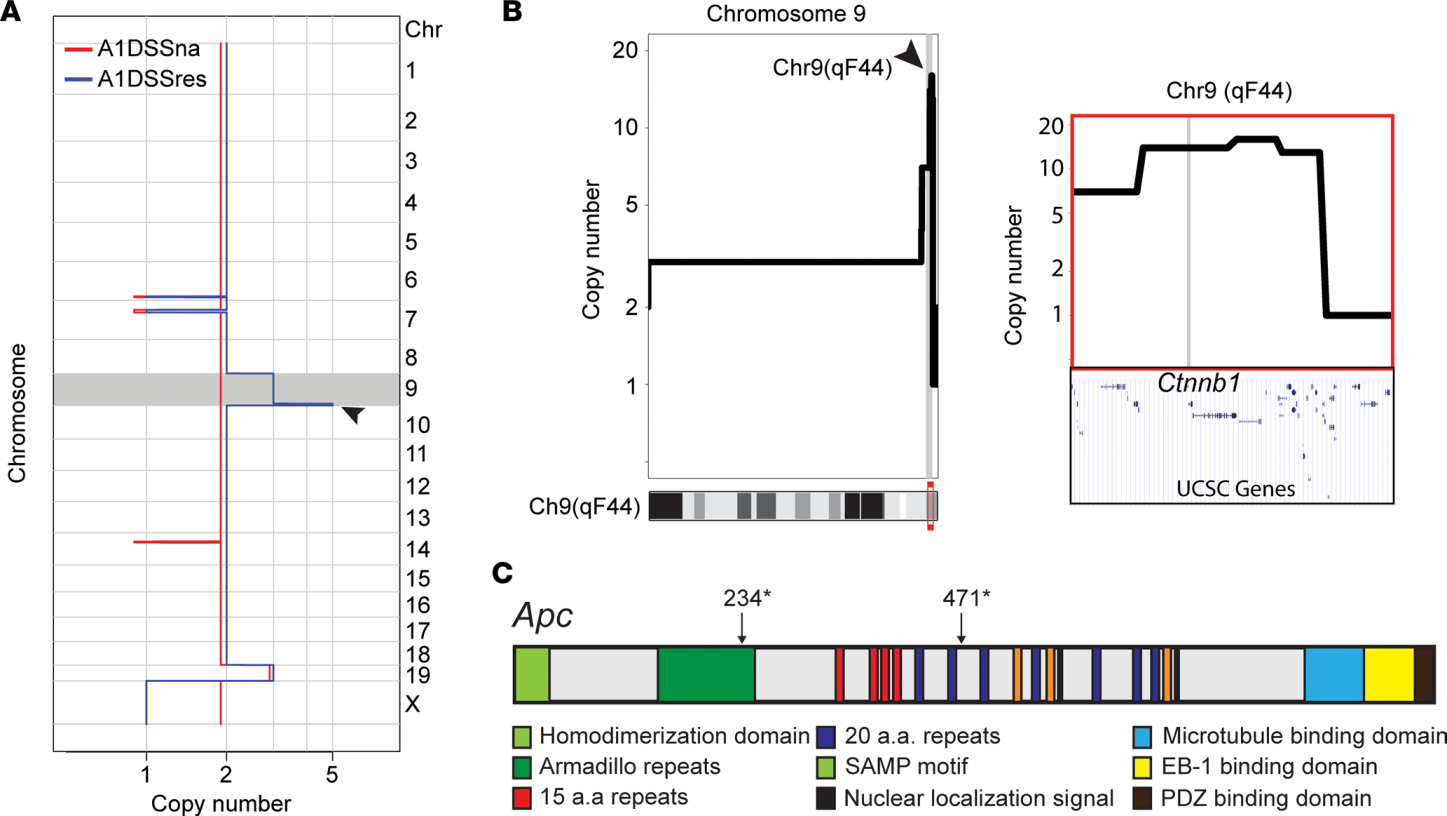
Niche disruption selects cells with oncogenic alterations conferring Wnt signaling pathway autonomy. (**A**) Genome-wide copy number profiles of A1DSSna and A1DSSres showing a focal amplification on chromosome 9. (**B**) Zoomed-in view of chromosome 9 shows high-level amplification of an approximately 1.3 Mb region on qF44 (left panel), a region that contains the genetic locus of *Ctnnb1* (encoding β-catenin) (right panel). Arrowheads in **A** and **B** point to a focal amplification on chromosome 9 in A1DSSres organoids. (**C**) Schematic representation of the mouse *Apc* gene, which identifies nonsense mutations Q234* and Q471* in the Apc protein domains: Armadillo repeats and 20 amino acid repeats, respectively, created using data from ref. 64.

Aberrant activation of the Wnt signaling program is the major driving force for initiation of human colorectal carcinogenesis (50). Expansion of the Lgr5^+^ cell compartment ensues. Gain of Wnt autonomy in A1DSSres and A2DSSres upon in vitro DSS selection suggests similar genetic drivers might mediate Wnt autonomy. To test this hypothesis, we initially employed whole-genome sequencing to investigate copy number alterations (CNAs), which revealed an overall flat copy number profile without large gains or losses of DNA. Intriguingly, A1DSSres organoids displayed amplification of about 1.3 Mb on chromosome 9 (Figure 6A). This region shows a focal increase of approximately 16 copies and includes the β-catenin gene (Figure 6B). Amplification of β-catenin has been reported in gastric cancer coinciding with enhanced nuclear β-catenin localization (60), and Lgr5^+^ chief cells were identified as a key cell of origin in early gastric cancer (61).

Subsequent in-depth analysis of genetic changes was performed using the custom-generated murine Memorial Sloan Kettering Integrated Mutation Profiling of Actionable Cancer Targets (MSK-IMPACT). Currently this assay analyzes all exons, and selected introns corresponding to common oncogenic rearrangements, of 578 known cancer-relevant mouse genes (62, 63). Using this approach, several key regulators of the Wnt pathway were found to be affected. In A2DSSna and A2DSSres organoids, the Apc gene carried 2 nonsense mutations (Q234*, Q471*). These mutations are found in the Armadillo repeats (Q234*) and in the 20 amino acid repeats (Q471*; Figure 6C) (64) and correspond to known driver mutations reported in human CRC (65, 66). These data imply that the CRC driver mutations were dormant in organoids from AOM-treated mice but were selected by DSS treatment under conditions of dysplasia and Lgr5^+^ stem cell expansion.

Additional mutations were found that might affect colorectal carcinogenesis. MSK-IMPACT analysis of A1DSSna and A1DSSres organoids identified potentially novel missense mutations in Apc, Gsk3β, and Axin2, key negative regulators of β-catenin (Supplemental Table 1). Beyond genetic changes in the canonical Wnt pathway, mutations in the tumor suppressor genes Trp53 and Nf1 and in the Pik3ca oncogene were also detected. Furthermore, chromatin remodeler Arid1a, histone acetylase Crebbp, DNA methylase Dnmt3a, and histone methylases Kmt2b and Kmt2d were also affected by protein-altering mutations (67). Thus, AOM administration causes a multitude of mutations, including known driver mutations in Wnt signaling, which are dormant until niche integrity is compromised by DSS, even in the absence of an inflammatory environment. Based on these observations, we posit that niche separation, caused by in vitro DSS treatment, selects for tumor-like clones autonomous for Wnt signaling.

### DSS directly affects tight junction organization.

**Figure 7:**
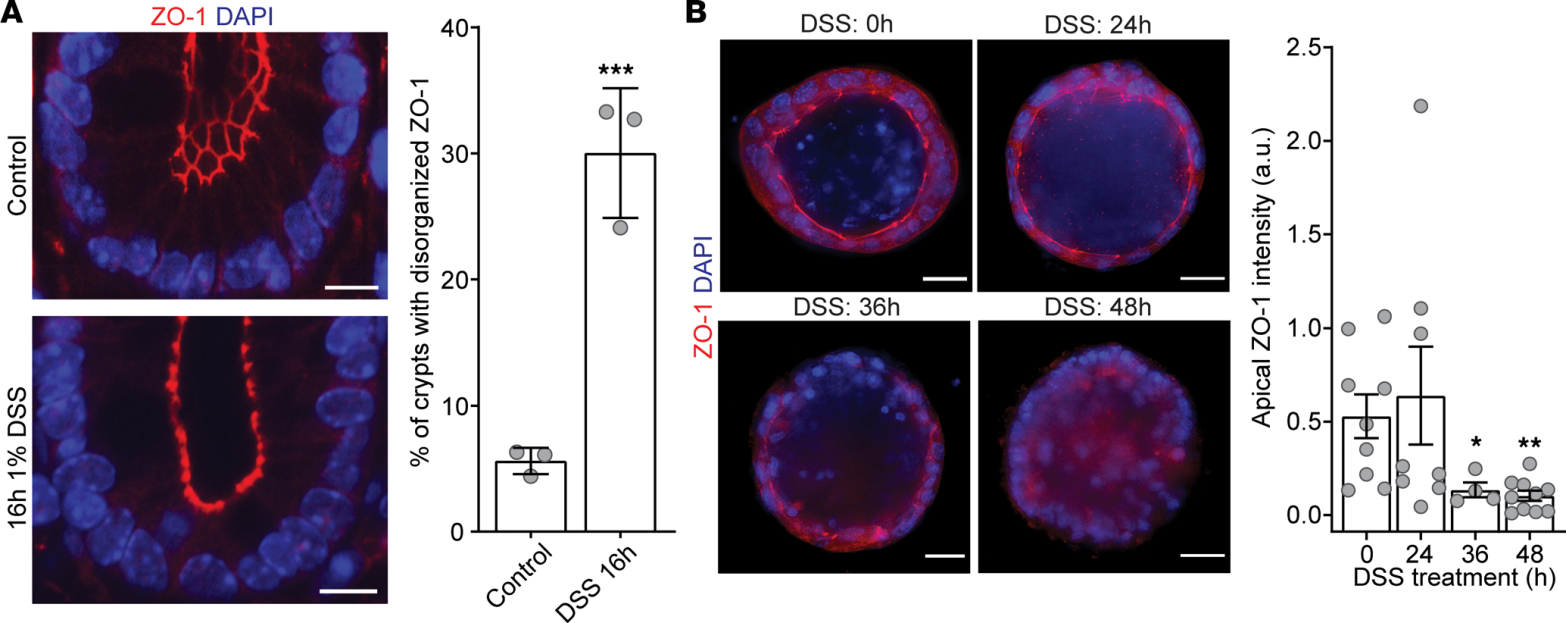
DSS disrupts localization of the tight junctional protein ZO-1 in vivo in the distal colon and in vitro in organoids derived thereof. (**A**) The left panel shows representative images of the impact of DSS on ZO-1 tight junctional localization in distal colonic specimens isolated from untreated control and 16-hour DSS-treated mice. Scale bar = 10 μm. The right panel quantifies (mean ± SD) the effect of 1% DSS on ZO-1 distribution evaluating 10–15 crypts/mouse in 3 mice/group. *P* < 0.002 (***), 2-tailed Student’s *t* test. (**B**) The left panel shows DSS (3 μg/mL) treatment of distal colon–derived LI2 organoids causes apical loss of ZO-1 over time (0–48 hours). Scale bar = 20 μm. The right panel quantifies this effect (mean ± SEM) showing significant difference in loss of ZO-1 between 0-hour untreated controls and 36-hour and 48-hour DSS treatment. Total *n* = 31 organoids. *P* < 0.02 (*), *P* < 0.01 (**), 2-tailed Student’s *t* test with Bonferroni’s correction.

A tight seal between neighboring epithelial cells is essential for optimal barrier function (68), but treatment with 1% DSS induces rapid separation of Lgr5^+^ CESCs from neighboring cKit^+^ niche cells in intact crypts (Figure 2A). DSS-induced separation of stem and niche cells is also accompanied by disruption of the highly ordered reticular pattern of the tight junction (TJ) protein zona occludens-1 (ZO-1) that outlines apical cell-cell contacts at the crypt base (*P* < 0.001, Figure 7A). Concomitant disorganization of crypt claudin-1 localization was also detected at the TJ (Supplemental Figure 8). To investigate the effect of DSS on ZO-1 ex vivo, DSS time course experiments were performed using normal LI2 wild-type large intestinal organoids treated for 0, 24, 36, and 48 hours with 3 μg/mL DSS in WENR. Organoids were stained as whole mounts using an immunofluorescent antibody for ZO-1 (Figure 7B, left panel). Apical ZO-1 TJ localization progressively diminished over 48 hours of treatment (Figure 7B, right panel), albeit slightly delayed as compared with in vivo treatment. These studies suggest that DSS directly affects integrity of large intestinal TJs concomitant with rapid separation of CESCs from other structural elements of the stem cell niche, a condition that may render WNT deprivation a driving force for selection of mutated CRC stem cells. Of note, we did not find evidence of nuclear ZO-1 or claudin-1 enrichment upon DSS treatment in mouse colonic crypts or in organoids, as previously described in nephron tubular cell lines, in terminally differentiated cells at the villus tip of mouse intestinal crypts, and in advanced colon cancer (69, 70).

### Resistance to niche disruption develops during normal human colon transition to adenoma.

**Figure 8:**
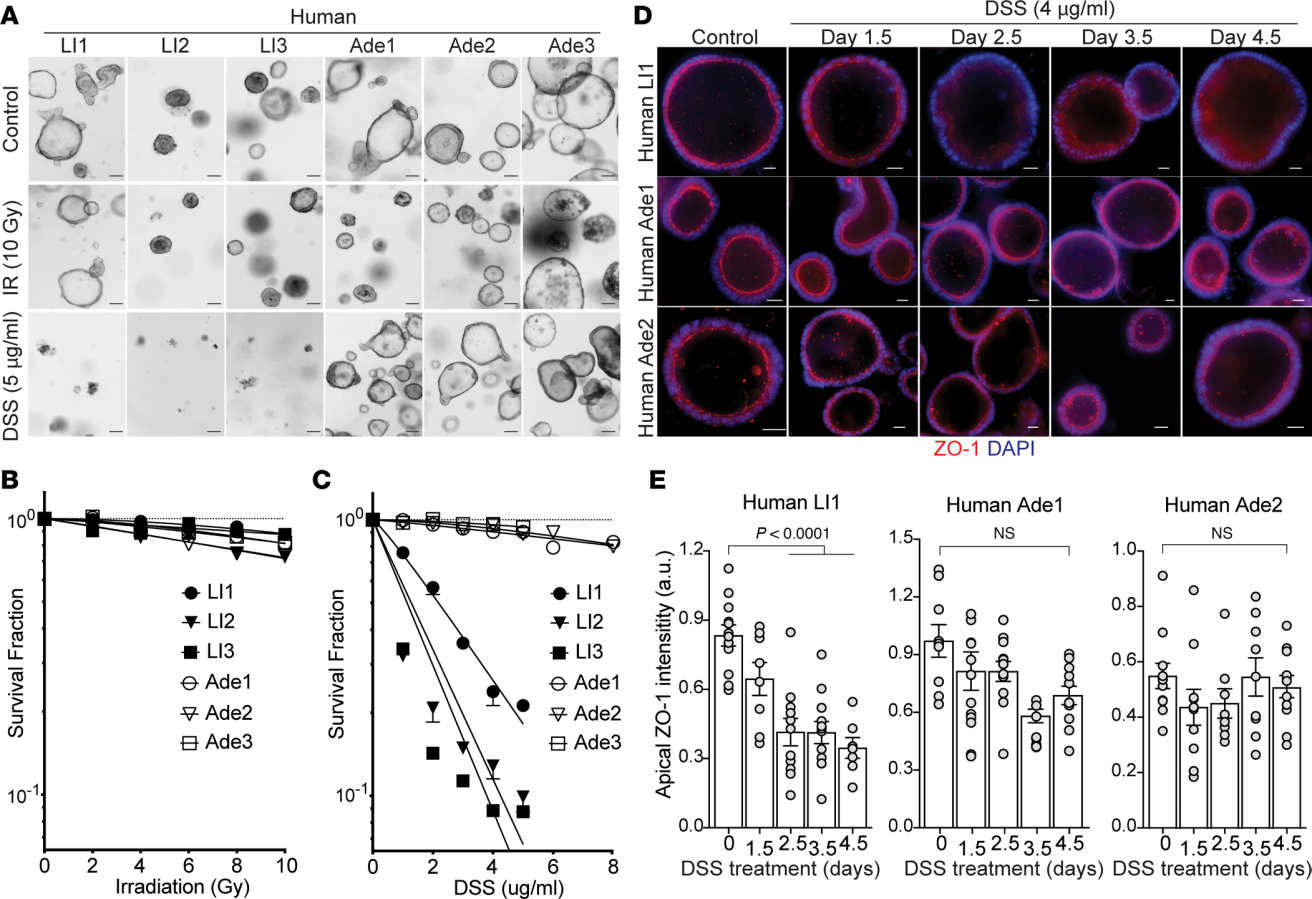
Human adenomas develop resistance to DSS-induced niche disruption during normal colon to adenoma transition. (**A**) Representative bright-field images of human LI1, LI2, LI3, Ade1, Ade2, and Ade3 organoids left untreated (control) or treated with either 10 Gy irradiation (IR) or 5 μg/mL DSS (day 7 data shown). Scale bar = 200 μm. (**B** and **C**) Dose survival curves reveal that while radiation resistance is unchanged during normal human colon to adenoma transition, normal human colon organoids are highly sensitive to niche disruption with DSS. Data (mean ± SEM) are from organoids treated with 0–10 Gy (**B**) or 0–8 μg/mL DSS (**C**) for 7 days. (**D**) Fluorescent ZO-1–stained images of DSS-treated (4 μg/mL) human normal (LI1) organoids for 1.5–4.5 days show time-dependent reduction of apical ZO-1, whereas human adenoma (Ade1 and Ade2) organoids are unchanged. Scale bar = 20 μm. (**E**) Quantification of images in **D** shows significant time-dependent loss of apical ZO-1 in normal human LI1 organoids but not in human Ade1 or Ade2 adenoma organoids. Data (mean ± SEM) are collated from more than 50 organoids/patient. Tukey’s honest significance test was used to generate *P* values due to multiple comparisons.

To test whether transition of normal human colon to adenoma features niche disruption, we leveraged a set of patient-derived organoids (PDOs) from our ongoing clinical trial (Memorial Sloan Kettering Cancer Center [MSKCC] Institutional Review Board Protocol 15-191, principal investigator [PI]: PBP) in which we compare impact of neoadjuvant chemoradiation on CRC in situ with organoids derived thereof. In this study, PDOs were generated from primary colorectal adenomas and carcinomas, and cognate normal colon mucosa (at least 5–10 cm from the tumor margin), in a cohort of patients undergoing the “watch and wait” protocol, a new strategy designed to closely follow potentially curable patients rather than uniformly perform colectomy (Hsu et al., manuscript in preparation). Using our clonogenic assay with SHMT analysis as above, we compared impact of 2 distinct lethal injuries on survival of normal human colon (LI1, LI2, LI3) and adenoma (Ade1, Ade2, Ade3) PDOs established before neoadjuvant therapy. Whereas PDOs from normal human colon and adenomas displayed comparable radiation-resistant profiles with D_0_ (Gy) values of LI1 = 24.6, LI2 = 30.8, LI3 = 37.2, Ade1 = 21.8, Ade2 = 28.0, and Ade3 = 31.9 (Figure 8, A and B), suggesting that the DNA damage response remains largely unchanged during normal colon to adenoma transition, these same organoids, like mouse organoids, displayed distinctly different DSS sensitivities. While normal human colon PDOs were highly sensitive to DSS treatment, displaying D_0_ (μg/mL) values of LI1 = 2.7, LI2 = 1.8, and LI3 = 1.6, human colorectal adenomas were without significant death up to 8 μg/mL DSS, displaying 11-fold increased D_0_ values of Ade1 = 29.6, Ade2 = 17.8, and Ade3 = 23.4 (Figure 8, A and C). Further, development of DSS resistance correlated with niche integrity. As with normal mouse organoids, normal human organoids displayed time-dependent reduction in apical ZO-1, with more than 50% decrease of the ZO-1 fluorescence signal by day 2.5 after 4 μg/mL DSS treatment. In contrast, human adenoma organoids retained ZO-1 localization at the apical TJ under the same DSS treatment (Figure 8, D and E). These data thus demonstrate differential alteration in response to these 2 death signals during progression of normal mucosa to adenoma. Altogether, our mouse and human organoid data support the notion that early-onset CRC tumorigenesis may initiate by niche dysregulation in the absence of inflammation.

## Discussion

**Figure 9:**
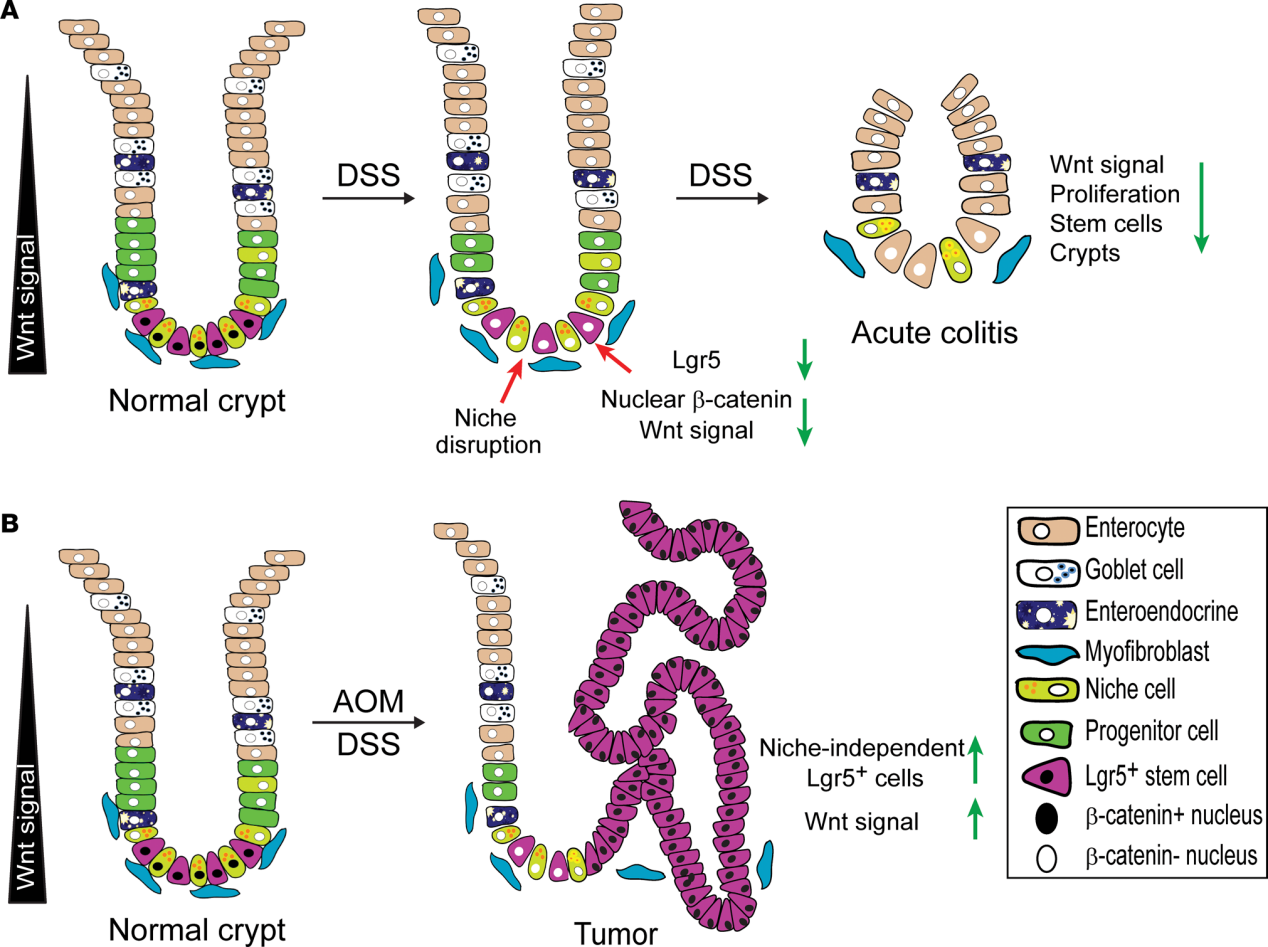
Schematic representation of the molecular mechanism proposed for colon tumorigenesis in the colitis-associated cancer model. (**A**) DSS physically disrupts interaction between Lgr5^+^ CESCs and cKit^+^ cells within hours, selectively degrading Wnt-driven stem cell function, leading to CESC dormancy and colitis induction. (**B**) However, in colonic tissue of mice pretreated with the mutagen AOM, stem cells escape inactivation of the stem cell program, acquiring gain-of-function mutations in the Wnt program and niche-regulatory function independence, predisposing to colon tumor formation.

The AOM-DSS model used in the current investigations conforms to the 2-stage in vivo model of carcinogenesis in which a mutagen (AOM) is alone insufficient for tumor outgrowth without application of a second nonmutagenic signal, a tumor promoter, that stimulates outgrowth of a mutated tumorigenic clone. Here we use organoid technology to separate the 2 stages in time and space to permit incisive examination of the properties of promotion that allow for outgrowth of a mutated clone from a seemingly normal mucosa. The colonic mutagen AOM induced genetic alterations in epithelial cells at random, but in an intact stem cell niche with normal Wnt signaling, mutated CESCs appeared not to have a fitness advantage over wild-type CESCs, and therefore were incapable of taking over the crypt. Our results reveal, however, that when niche integrity was compromised by DSS treatment, wild-type CESCs, dependent on exogenous Wnt signaling for homeostasis, became dormant, whereas mutated CESCs, autonomous for intracellular Wnt signaling, continued proliferating. The net outcome of DSS disruption of normal niche function was selection for a mutated clone that irreversibly dominated the organoid, comparable to the observation that repeated introduction of DSS in the drinking water of an AOM-treated mouse resulted in irreversible tumor formation in vivo (Figure 9). This mechanism is reminiscent of the concept underlying “adaptive oncogenesis” proposed by DeGregori and colleagues, which argues that the fitness of mutant stem cells becomes more competitive as integrity of the normal stem cell niche becomes degraded as the result of aging or other disruptions (71, 72).

DSS-induced separation of CESCs from the mesenchymal Wnt source presented by basal lamina in vivo is presumably recapitulated ex vivo in organoid culture by induced separation of CESCs from the basement membrane surrogate provided by Matrigel, as well as from growth signals such as EGF and Notch provided by cKit^+^ colonic niche cells. Resulting outgrowth of CESCs displaying 2 different Wnt gain-of-function programs, both of which have driver counterparts in human GI cancers, is a testimony to the power of organoid technology to serve as a model system to study the process of tumorigenesis in the absence of a systemic circulation and immune system. While this set of events may be critical for initiating tumor formation, a large body of literature indicates that tumor formation is greatly enhanced by inflammation. Physical separation of the niche, in addition to loss of Wnt, simultaneously leads to attenuated barrier function, allowing infiltration of pathogens followed by an immune response (73). This inflammatory response has profound impact on extent and number of tumors formed (10, 13, 20–22). Several inflammatory signaling pathways, such as NF-κB, JAK/STAT, and PI3K/AKT, provide prosurvival and proliferative signals to developing CRC cells, and genetic inhibition of these pathways uniformly reduces inflammation and tumor number (20, 22, 74). In the context of the AOM-DSS model, deletion of STAT3 in colonic epithelium reduces the number and size of adenomas, yet the AOM-DSS–challenged colon still shows multifocal flat low-grade intraepithelial neoplasia (20). This is consistent with the concept that niche separation serves dual functions, loss of local WNT signals leading to selection of a mutated clone and also inflammation-induced tumor progression.

Recognition of this dual mechanism of tumor formation evolving from niche disruption prompts a reconsideration of the AOM-DSS model of colorectal carcinogenesis. Development of human CRC is influenced by a variety of genetic, environmental, and inflammatory factors. Genetic factors include rare hereditary syndromes such as adenomatous polyposis coli (germline APC mutation) and Lynch syndrome (75). While the vast majority of CRCs are sporadic, a small subset of patients with IBD are at enhanced risk of developing CRC, resulting in the clinical strategy of prophylactic colectomy (76). While both sporadic and colitis-associated CRC have a common pathologic fate, their pathogenesis is distinct. Most sporadic CRCs follow the known Vogelstein paradigm of adenoma to carcinoma transition, with adenoma being the predominant pathologic precursor. This sequence, however, is largely absent in most colitis-associated CRCs, as dysplasia is the predisposing lesion to frank cancer in the majority of patients, while adenomas are rarely found in early-stage colitis-associated carcinogenesis (77). These differences in cancer progression are reflected in differences in genetic alterations. While mutations that aberrantly activate the Wnt pathway (e.g., APC) are found in over 85% of sporadic CRCs (78), in colitis-associated CRC mutations in the Wnt pathway are far less frequent (79). Furthermore, detailed analysis of the kinetics of acquisition of the mutational profile indicates that APC mutations are one of the earliest events in sporadic CRC pathophysiology (42), whereas in colitis-associated CRC, APC mutations occur later at the stage of high-grade dysplasia (80). For p53 mutations, the sequence is reversed, with p53 loss of heterozygosity as an early event in colitis-associated CRC and a much later event in the adenoma-carcinoma sequence of sporadic CRC (77, 80).

While AOM-DSS–induced colorectal tumor formation has been widely discussed as a model of inflammation-induced tumorigenesis, our data showing tumor progression ex vivo in the absence of a humoral immune system (and the above data relating tumor progression through adenoma transition and selection of early APC mutations) suggest that the AOM-DSS model should be considered more as a model of sporadic carcinogenesis. Reminiscent of the sporadic human disease, it appears possible to convert AOM-DSS adenomas into carcinomas ex vivo as AOM-DSS adenoma organoids isolated from mice harboring a latent gain-of-function *Kras^tm4tyj/+^* allele treated with Cre recombinase (81) develop a phenotype highly similar to organoids generated from human CRCs in our laboratory (Hsu, Adileh, and Kolesnick, unpublished observation).

Extension of these concepts in the current study to human organoids suggests development of resistance to niche injury represents a hitherto unknown feature of the adenoma phenotype, at least for sporadic CRC. Further, recognition that the AOM-DSS model represents a model of sporadic human CRC coupled with judicious use of organoid technology could provide an experimental platform to study important issues in CRC difficult to study using current methodologies. For instance, organoids from AOM-treated mice would permit closer investigation of agents that might phenocopy DSS, such as dietary components or food additives that might potentially trigger onset of tumorigenesis by introducing colonic niche injury (82, 83), or the identification of biologic and pharmacologic tools that might limit outgrowth of mutated clones ex vivo, concepts currently being pursued using this technology in our laboratory.

## Methods

### Mice.

*Lgr5-lacZ*, *Lgr5-EGFP-ires-CreERT2*, *Lgr5-EGFP-ires-CreERT2/Rosa26-lacZ* (gifts of Hans Clevers, Utrecht University, Utrecht, the Netherlands), and *Lgr5-ires-CreERT2(3*′*UTR)/Rosa26-mTmG* strains (gift of Tuomas Tammela, MSKCC) were genotyped and used as described (2, 31).

### Acute colitis and tumor induction.

The colitis protocol was based on preliminary toxicity data in the *Lgr5-lacZ* strain. The standard protocol to induce acute colitis uses 2%–3% DSS for 7 consecutive days dependent on mouse strain sensitivity (84). As we found approximately 30%–40% of *Lgr5-lacZ* mice died at day 6 or 7 after DSS treatment because of severe colon damage at ≥2% DSS, we elected to use 1% DSS in drinking water ad libitum (MP Biomedicals or TdB, molecular mass 36–50 kDa) for colitis induction in this strain. Adenoma induction used a standard AOM-DSS protocol (21, 84) modified as follows: electing a schedule of decreasing DSS dosage to mitigate toxicity, 6- to 8-week-old male *Lgr5-lacZ* mice were injected intraperitoneally with AOM (MilliporeSigma) at a dose of 12.5 mg/kg body weight and after 5 days were fed 2% DSS in the drinking water for 5 days, followed by 14 days of regular water. This cycle was repeated twice with 1.5% DSS and 1.0% DSS, respectively. Colonoscopy was performed at day 90 on anesthetized mice to check for tumor formation using the Coloview System (Karl Storz). Our data show a single AOM injection does not induce colon tumorigenesis in C57BL/6J mice (data not shown) (40, 85, 86).

### Crypt isolation and organoid culture.

Crypt isolation and culture were performed as described (38, 48) with minimal modification. Briefly, distal colon was removed and flushed with cold PBS (Gibco, Thermo Fisher Scientific) containing 100 U/mL penicillin and 100 U/mL streptomycin (Gibco, Thermo Fisher Scientific). Intestinal fragments or adenomas were sliced into 1 mm^3^ pieces, then suspended into 10 mL of DMEM (Gibco, Thermo Fisher Scientific) containing 1% FBS and 500 U/mL collagenase IV (MilliporeSigma). The mixture was incubated for 30 minutes at 37°C in a shaking water bath. Thereafter, tissue fragments were shaken vigorously using a 10 mL pipette to isolate crypts, then allowed to settle under gravity for 1 minute, and the supernatant was collected for inspection by inverted microscopy (Nikon Eclipse TS100). The resuspension/sedimentation procedure was repeated 4 times. Liberated crypts/crypt-like structures (from adenomas) in suspension were combined, passed through a 100 μm cell strainer (BD Biosciences), and then centrifuged at 4°C at 300*g* for 5 minutes. The pelleted crypts/crypt-like structures were washed with cold Advanced DMEM/F12 (ADF; Gibco, Thermo Fisher Scientific), then centrifuged at 60*g* for 3 minutes to separate crypts from single cells. After centrifugation, crypts/crypt-like structures were counted and resuspended in Matrigel (Corning) covered with ADF medium containing 1 mM HEPES, 1 mM Glutamax, and 100 U/mL antibiotics, supplemented with 1× B27, 1× N-2 (all Invitrogen), as well as *N*-acetylcysteine (1 mM, MilliporeSigma), murine recombinant EGF (100 ng/mL, PeproTech), and conditioned media from 50% Wnt3a (v/v) and 10% R-spondin1 (v/v) and 5% Noggin (v/v, designated as WENR) as described (43, 48). Conditioned media were collected as described (6, 43, 48).

### In vitro tumor selection.

For in vitro adenoma formation, *Lgr5-lacZ* and *Lgr5-EGFP-ires-CreERT2* mice were injected intraperitoneally with AOM at 10 mg/kg body weight weekly for 5 weeks. At 9–12 weeks after the last injection, crypts were isolated as described above and cultured in WENR. Over several passages, 2 organoid lines from AOM-treated mice were treated with increasing doses of DSS in increments starting at 1 μg/mL DSS until organoids tolerated a dose of 2.5 μg/mL DSS. At this point, Wnt3a-conditioned media concentration was lowered incrementally from 50% to 1% Wnt3a (final volume). Parental organoids from AOM-treated mice, before DSS selection (DSS naive) were named A1DSSna or A2DSSna, and selected lines (DSS resistant) were termed A1DSSres or A2DSSres. During passaging, organoids were physically disrupted, and organoid fragments were separated from single cells by 60*g* centrifugation for 3 minutes at 4°C. Overnight, organoid fragments recovered without DSS, followed by addition of the indicated DSS concentration the following day.

### PDOs.

Normal human colonic tissue and adenomas from patients were collected at time of surgical resection or endoscopic examination at MSKCC, and the diagnosis was confirmed by the Pathology Core. Isolation and culture were as described above with the following additions: 10 nM gastrin (MilliporeSigma), 500 nM A83-01 (Tocris), 10 mM SB202190 (MilliporeSigma), 10 mM nicotinamide (MilliporeSigma), and 50 ng/mL human recombinant EGF (PeproTech). To increase PDO survival, 10 μM of the ROCK inhibitor, Y27632, was added during the first 3 days of culture. Normal human colon PDOs were grown in WENR medium, whereas human adenoma PDOs were maintained in EN medium without Wnt and R-spondin1. For the irradiation experiment, detailed methodology and the mathematical algorithm fitted to the SHMT model were as described (47, 87). Briefly, PDOs were plated at a density of approximately 150 organoids/well in triplicate per dose. On the day after plating, PDOs were exposed to single-fraction radiation ranging from 0 to 10 Gy using a Shepherd Mark-I unit (Model 68, SN643, J. L. Shepherd & Associates) operating ^137^Cs sources at 1.72 Gy/min. Surviving organoids at day 6 after radiation were counted under bright-field microscopy to generate classic radiation dose survival curves, in which surviving fraction was defined as number of surviving organoids/number of organoids in unirradiated controls.

### Wnt3a and DSS dose-response curves.

Organoids were split as described above. The following day, organoids were treated with media containing different growth factor compositions: group 1: WENR; group 2: EGF (100 ng/mL), 5% Noggin, and 10% R-spondin1 (ENR); group 3: EGF and Noggin (EN). Growth of organoids was followed for 7 days, and on day 1, 3, 5, and 7, images were taken on BioTek Cytation 5 and analyzed for growth using ImageJ (FIJI). On day 7 surviving organoids were quantified. DSS dose-response experiments were carried out in WENR supplemented with respective concentrations of DSS and analyzed as described above.

### Staining for β-galactosidase (lacZ).

*Lgr5*-*lacZ* mice were euthanized and a 3 cm segment of distal colon stained for presence of β-galactosidase as done before (2, 30).

### Histology, immunofluorescence, immunohistochemistry, and ZO-1 whole mount staining.

Paraffin-embedded, 5 μm sections of distal colon were deparaffinized, rehydrated through graded ethanol, and stained with H&E for morphologic analysis. Primary antibodies were rabbit polyclonal anti–chromogranin A (ab15160, Abcam, 1:400), rabbit polyclonal anti–ZO-1 (61-7300, Zymed, 1:200), rabbit polyclonal anti-mucin2 (SC-15334, Santa Cruz Biotechnology, 1:200), goat polyclonal anti-cKit (AF1356, R&D Systems, 1:200), rabbit anti–caspase-3 (9661, Cell Signaling Technology, 1:100), and rat monoclonal anti–mouse/human CD44 (103001, BioLegend, 1:100). Secondary antibodies were biotin conjugated and visualized using the Vectastain Elite ABC-HRP Peroxidase kit (PK-6100; Vector Laboratories). Frozen sections (5 μm) of distal colon were fixed in 4% paraformaldehyde for 10 minutes and then stained for claudin-1 (rabbit polyclonal anti–claudin-1, catalog 71-7800, Zymed, 1:200). Staining for β-catenin (BD Biosciences, catalog 610153, 1:100) was performed as described (35). For organoid staining, paraffin-embedded, 5 μm organoid sections were handled as described (47) and stained as for distal colon above. For quantification of single versus multiple cell layers, each organoid was divided in half, and number of cell layers in each half was counted independently. To minimize sectioning artifacts, at least 300 organoids from 3 individual experiments were evaluated. For the *Lgr5-mTmG* lineage tracing experiment, age- and sex-matched mice were intraperitoneally injected with 0.5 mg/100 μL of 4-OHT (MilliporeSigma). After 4 days of induction, mice were randomly separated into 2 groups (3 mice/group), 1 with and 1 without 1% DSS in their drinking water, for 2 days. After sacrifice, surgically removed colon was fixed in 10% formalin for 24 hours at 4°C, followed by incubation with 30% sucrose overnight at 4°C. Specimens were then OCT (Thermo Fisher Scientific) mounted and subjected to frozen sectioning (10 μm). Images were captured by a Zeiss Axio wide-field microscope or Leica TCS SP5 confocal microscope. For the ZO-1 whole mounting experiment, organoids were seeded onto 8-well Lab-Tek chambers (Nunc, Thermo Fisher Scientific) precoated with a thin layer of 100% Matrigel. The organoids were overlaid with WENR medium containing Matrigel (final 2%) and 3 μg/mL DSS (for mouse organoids) or 4 μg/mL DSS (for human organoids), then incubated at 37°C for indicated times. Immunostaining was performed using a standard protocol. In short, organoid cultures were fixed with 4% paraformaldehyde for 30 minutes and quenched with 50 mM NH_4_Cl for 20 minutes, followed by incubation with blocking buffer (PBS with 0.5% BSA, 0.02% Na-azide, 0.36 μM DAPI, and 0.25% Triton X-100) for 3 hours at room temperature (RT). Organoid samples were incubated with rabbit ZO-1 antibody overnight at 4°C followed by Alexa Fluor 594–conjugated secondary antibody for 2 hours at RT. Samples were mounted with ProLong Diamond Antifade reagent (Life Technologies). Nine consecutive 0.3 μm *Z*-sections (3 μm total) were imaged, deconvoluted using theoretical PSF using AutoQuant X software, and max-projected into a single image using ImageJ (FIJI). For organoid ZO-1 quantification, total ZO-1 intensity was measured using ImageJ (FIJI), lumen background signal was subtracted, and data were normalized to DAPI intensity in each organoid. Quantification of Lgr5 ISH was performed as follows: stained organoids were imaged with a scanning microscope (Miras Scanner) and analyzed using ImageJ (FIJI). For each organoid, total cell number and Lgr5^+^ cell number were counted, and percentage of Lgr5^+^ cells in each organoid was calculated. For organoid β-catenin quantification, we applied methods similar to those reported (54–57). Images were analyzed using FIJI and percentage of β-catenin^+^ cells per organoid was calculated.

### Electron microscopy analysis.

Two *Lgr5-lacZ* male mice, 6–8 weeks old, were used per time point. Colonic samples were fixed in 2.5% glutaraldehyde plus 2% paraformaldehyde in 0.075 M sodium cacodylate buffer for 1 hour at 4°C and postfixed in 2% osmium tetroxide in double-distilled water. Samples were then rinsed in double-distilled water and dehydrated in a graded alcohol series of 50%, 75%, and 95% through absolute alcohol, followed by propylene oxide, with overnight incubation in 1:1 propylene oxide/poly bed 812 epoxy resin (manufacturer unknown). Ultrathin sections, obtained with a Reichert Ultracut S microtome, were stained with uranyl acetate and lead citrate and photographed using a Jeol 1200 EX transmission electron microscope. Experiments were performed in the Electron Microscopy Facility at the Sloan Kettering Institute.

### EdU incorporation assay.

Eight male *Lgr5-lacZ* mice, 6–8 weeks old, were randomly divided into a control or DSS-treated group (1% DSS, 48 hours). Mice were injected intraperitoneally with 100 μL of 20 mM EdU solution 2 hours before sacrifice. Distal colons were collected for paraffin embedding and EdU staining. EdU was detected with the Click-iT EdU cell proliferation kit according to manufacturer’s instruction (Invitrogen).

### ISH.

ISH was performed using RNAscope 2.5HD (Advanced Cell Diagnostics) according to manufacturer’s recommendations, except for probe hybridization a ThermoBrite (Abbott Molecular) hybridization oven was used. The following probes were used: *Lgr5*: Mm-Lgr5: 312171; negative control: DabP: 310043; and positive control: Mm-Ubc 484311.

### MSK-IMPACT sequencing.

After PicoGreen quantification (Thermo Fisher Scientific), 100 ng of mouse genomic DNA was used for library construction using the KAPA HyperPrep Kit (Roche KK8504) with 8 cycles of PCR. After sample barcoding, 50–100 ng of each library was pooled and captured by hybridization with the M_IMPACT_v1 assay, which captures all protein-coding exons and select introns of 578 cancer-related genes. Capture pools were sequenced on the HiSeq 4000, using the HiSeq 3000/4000 SBS Kit (Illumina) for PE100 reads. Following these criteria, the average coverage was 569X, with an average of 99% of the targeted sequences covering 30X.

### Sparse whole-genome sequencing for copy number inference.

To determine CNAs at high resolution, multiplexed precapture libraries were sequenced to an average depth of 5 million sequencing reads. Absolute copy number calls were retrieved as previously described (88). In brief, sequencing reads were mapped, with uniquely mapped reads counted within genomic bins after normalization. Normalized read counts were subsequently segmented using CBS (89) and transformed to absolute copy number calls using a least squares fitting algorithm allowing copy number inference at a resolution of 300 kb (88).

### Statistics.

Values represent mean ± 95% CI, SD, or SEM. Differences were analyzed by 2-tailed Student’s *t* test or 1-way ANOVA. For human ZO-1 immunofluorescence analysis, Tukey’s multiple comparisons test was performed. Note that after consultation with ZZ at MSK, 4 observations much greater than 2–6 standard deviations from the mean were excluded from the human ZO-1 data analysis as a quality control, as they were considered outliers for such moderate samples. A *P* ≤ 0.05 was considered significant. Data were plotted using GraphPad Prism 8 software.

### Study approval.

All animal studies were approved by MSKCC Institutional Animal Care and Use Committee. All patients provided written informed consent before specimens were procured under MSKCC Institutional Review Board Protocol #15-191 (PI: PBP).

## Author contributions

SK, KSH, and GH contributed equally to this work. SK and GH conceived, designed, and initiated the study and performed most in vivo mouse experiments. SK and MLM developed organoid methodology in the laboratory. KSH established the PDOs and conceived, designed, and performed the human organoid experiments. KSH developed ZO-1 whole mount staining and performed the mouse lineage tracing experiment. TB analyzed and interpreted sequencing data. SK, KSH, GH, and MLM analyzed the data. ZZ assisted with statistical analysis. MA assisted with human organoid development. RK, AMCB, ZF, and PBP provided critical insights in experimental design and interpretation. SK, KSH, and RK jointly prepared the manuscript with input from all authors. PBP and RK secured funding and supervised the work.

## Supplementary Material

Supplemental data

## Version 1 03/08/2022

Electronic publication
