# Supplementary material for: Disruption of the crypt niche promotes outgrowth of mutated colorectal tumor stem cells
Source: JCI Insight. 2022 Mar 8;7(5):e153793. doi: 10.1172/jci.insight.153793 (PMC8983138; doi:10.1172/jci.insight.153793)
Supplement: Supplemental data [file jciinsight-7-153793-s231.pdf]

Supplementary Materials for

Disruption of the Crypt Niche Promotes Outgrowth of Mutated Colorectal Tumor Stem Cells

Stefan Klingler[1#+], Kuo-Shun Hsu[1#], Guoqiang Hua[2#], Maria Laura Martin[1‡], Mohammad Adileh[1], Taimour Baslan[3], Zhigang Zhang[4], Philip Paty[5], Zvi Fuks[6], Anthony M.C. Brown[7] and Richard Kolesnick[1§]

[§]Correspondence: E-mail: r-kolesnick@ski.mskcc.org

This PDF file includes:

Figure S1
Figure S2
Figure S3
Figure S4
Figure S5
Figure S6
Figure S7
Figure S8
Table S1

Figure S1

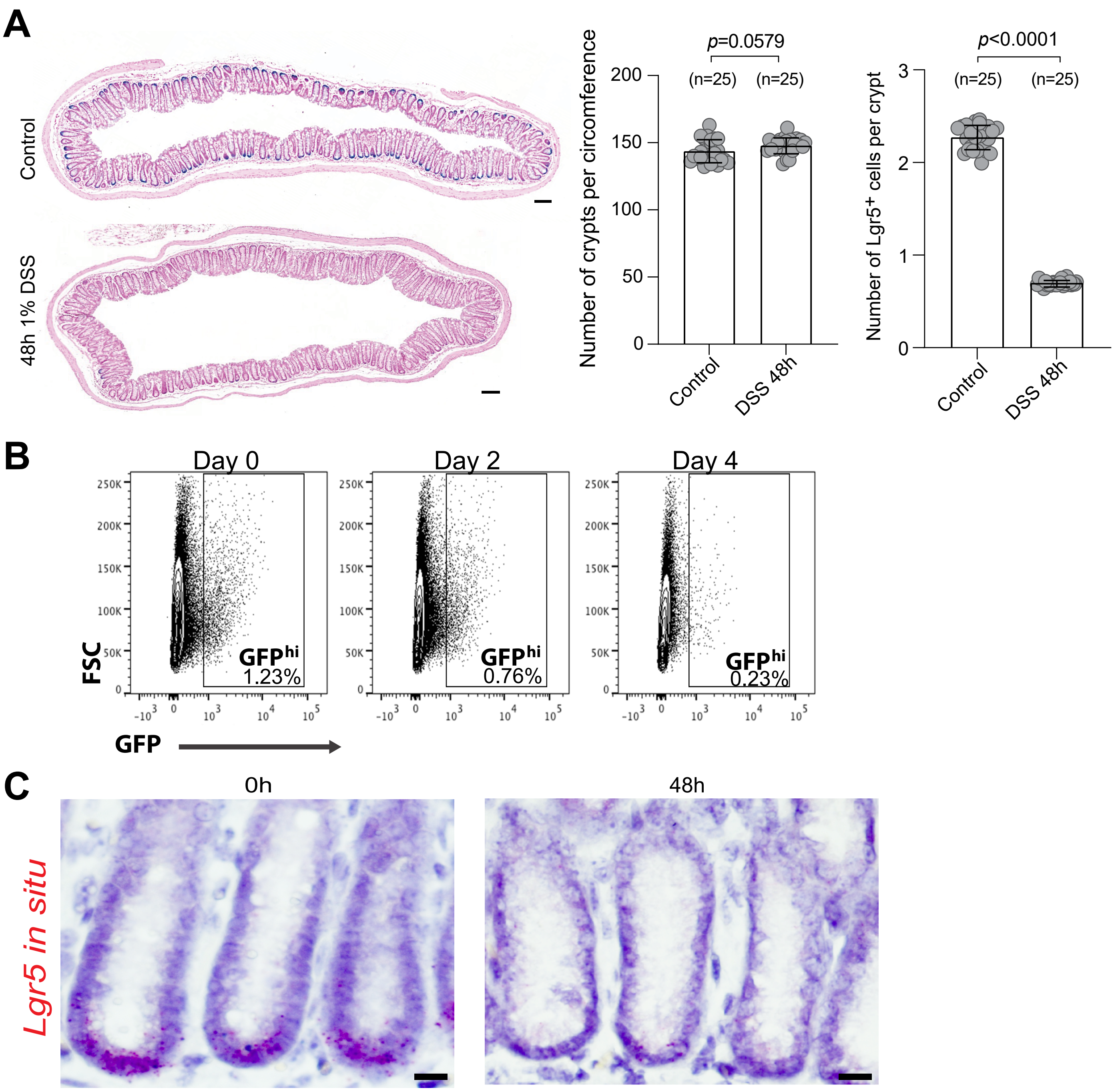

Figure S1. DSS induces loss of Lgr5 expression in CESCs, related to Figure 1. (**A**) Left panels - Representative transverse sections of distal colon from Lgr5-LacZ mice treated with or without 1% DSS for 48h and stained with LacZ. Middle panel - Quantification shows no difference in number of crypts per circumference with or without DSS treatment. For each group, 25 colonic circumferences from 3 mice were counted. Right panel - Number of Lgr5^{+} cells per crypt per section. Data presented as mean±SD, p value determined by Student's t-test. Scale bar = 100 µm. (**B**) Flow cytometry analysis of Lgr5 expression in crypt epithelium from distal colon of Lgr5-EGFP-ires-CreERT2 mice treated with 1% DSS at the indicated times. FSC, forward scatter. (**C**) Loss of Lgr5 expression was confirmed by Lgr5 ISH. Scale bar = 10 µm.

Figure S2

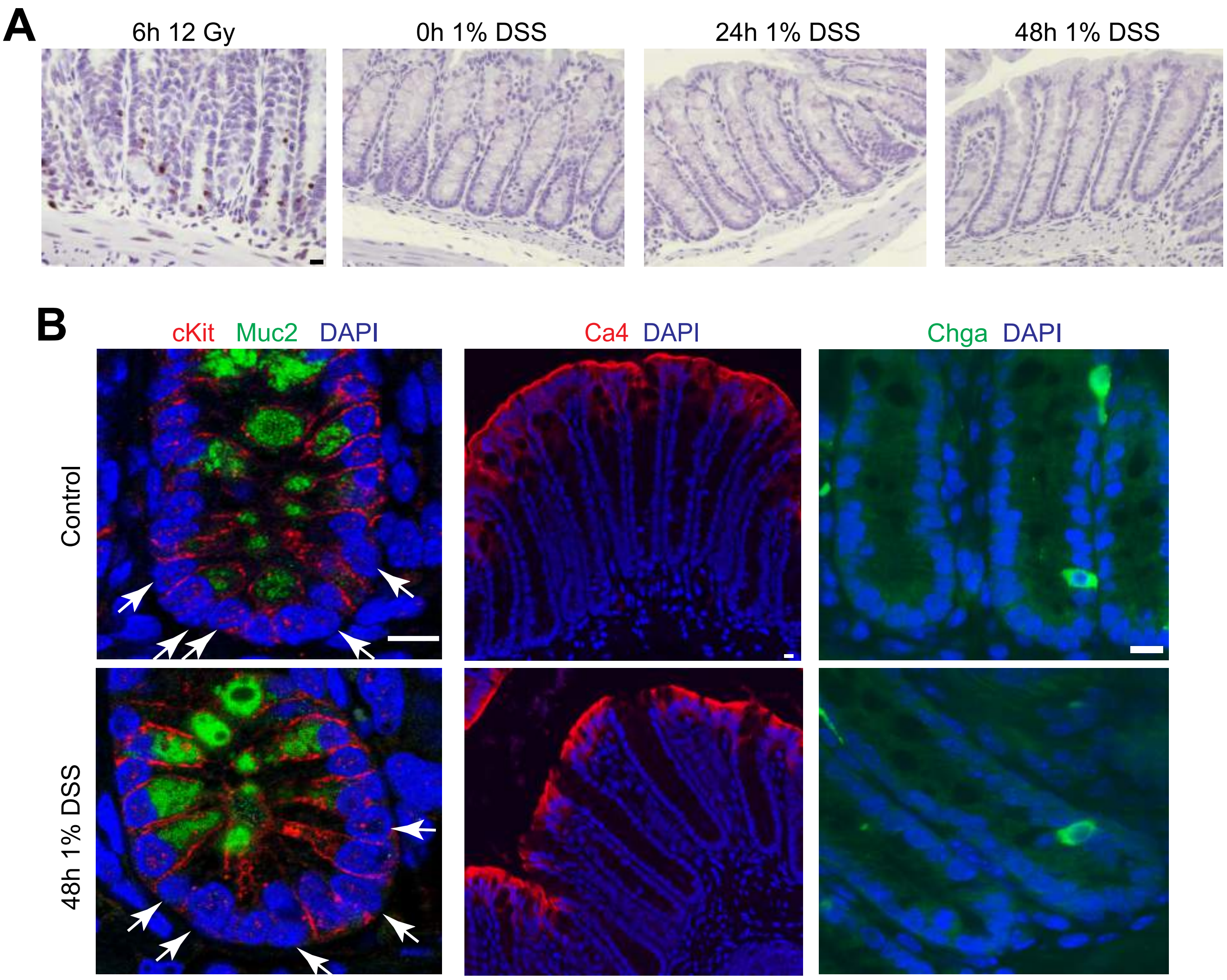

Figure S2. Apoptosis/differentiation is not the cause of DSS-induced loss of Lgr5 expression at the crypt base, related to Figure 1. (**A**) Lgr5-lacZ mice were treated with 1% DSS for 24h and 48h. Apoptosis was evaluated by staining for active Caspase3 fragment. Normal crypts irradiated with 12 Gy serve as positive control as published by us (30). Scale bar = 10 µm. (**B**) Immunofluorescence staining of Muc2 (for goblet cells), cKit, carbonic anhydrase IV (Ca4; for enterocytes) and chromogranin A (Chga; for enteroendocrine cells) in colonic crypts from Lgr5-lacZ mice treated with drinking water with or without 1% DSS indicates no differentia-tion occurred at the crypt base after DSS treatment. White arrows identify CESCs. Scale bar = 10 µm.

Figure S3

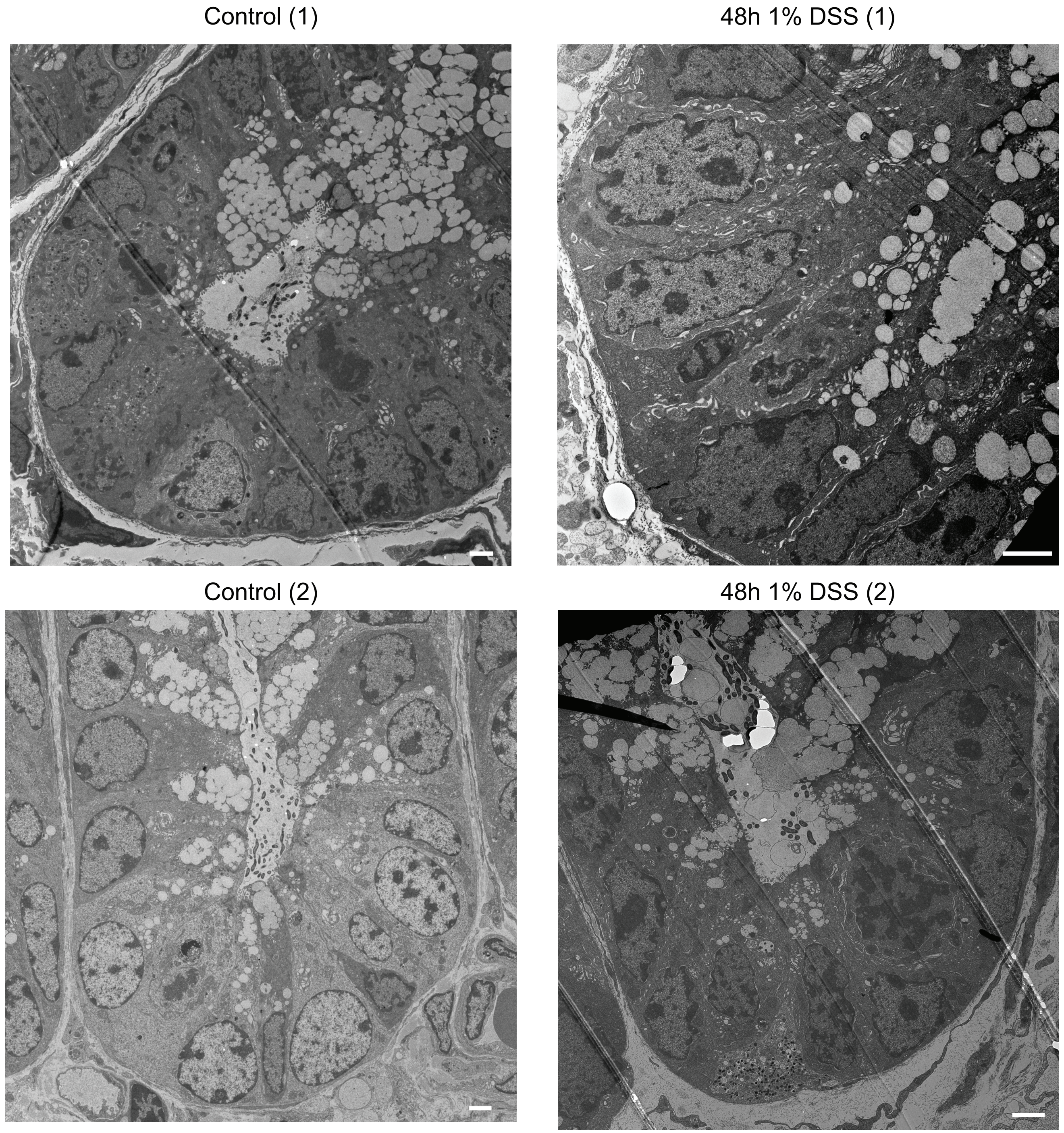

Figure S3. Uncropped EM images of mouse colonic crypts untreated or treated with 1% DSS for 48h, related to Figure 2. Two representative EM images are shown for untreated (control) and DSS-treated mouse colonic crypts. Scale bar = 2 μm.

Figure S4

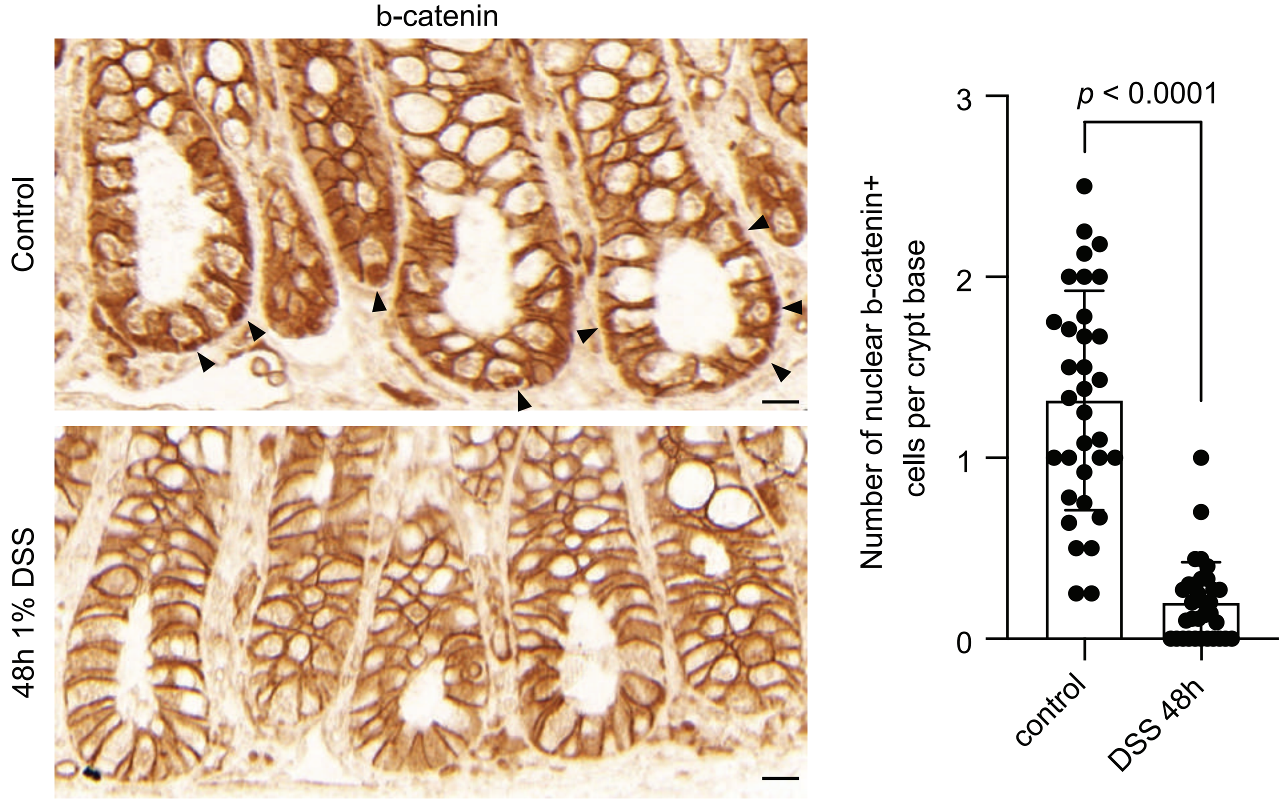

Figure S4. DSS induced niche disruption impairs nuclear translocation of β-catenin in colonic crypts, related to Figure 2. Left panels - Representative IHC images for nuclear β-catenin in mouse colon crypts untreated or treated with 1%DSS for 48h. Arrowheads identify cells with nuclear β-catenin signal at the crypt base. Scale bar = 10 μm. Right panel - Quantification of nuclear β-catenin^{+} cells per crypt base. Data represent mean±SD. In each group, 33 random fields of view from 3 mice were counted. Each data point represents number of nuclear β-catenin^{+} cells per total number of crypts per microscopic field. $p < 0.0001$; Student's t test.

Figure S5

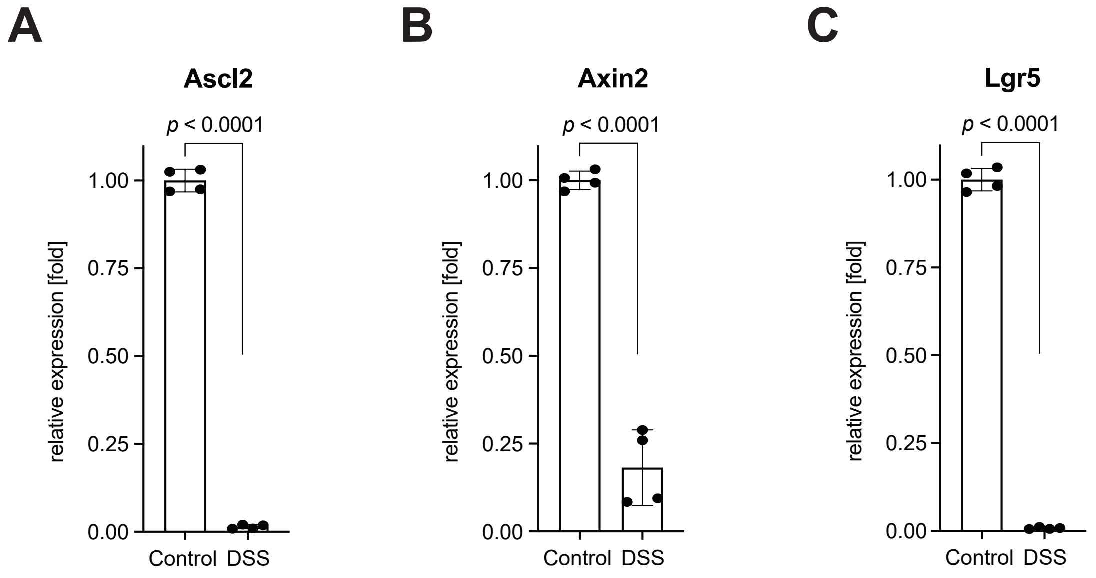

Figure S5. DSS treatment reduces expression of Wnt target genes, related to Figure 2. Expression of (**A**) Ascl2, (**B**) Axin2, and (**C**) Lgr5 genes in L1 wildtype organ-oids untreated or treated with 1% DSS for 48h was determinded by real-time PCR. Data repre-sent mean±SD collated from two independent experiments. *p* value, Student's t test.

Figure S6

Untreated

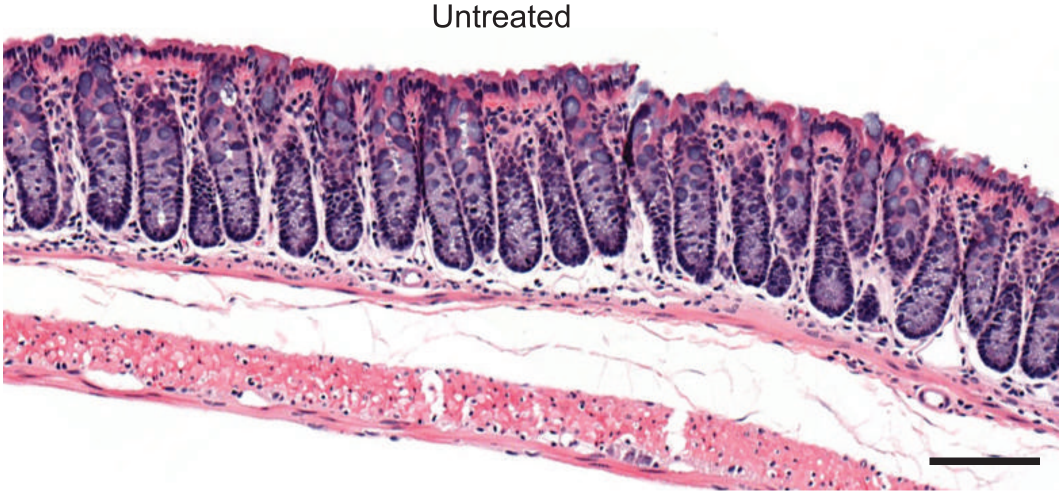

AOM

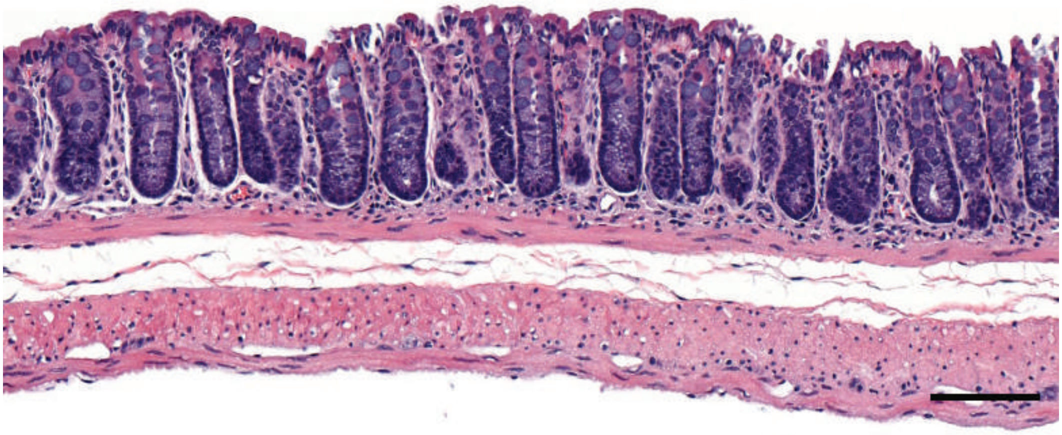

Figure S6. AOM treatment alone does not cause adenoma formation in C57BL/6 mice, related to Figure 3. Representative H&E images of an untreated C57BL/6 mouse and an AOM-treated C57BL/6 mouse show no abnormal phenotype. Scale bar = 200 µm.

Figure S7

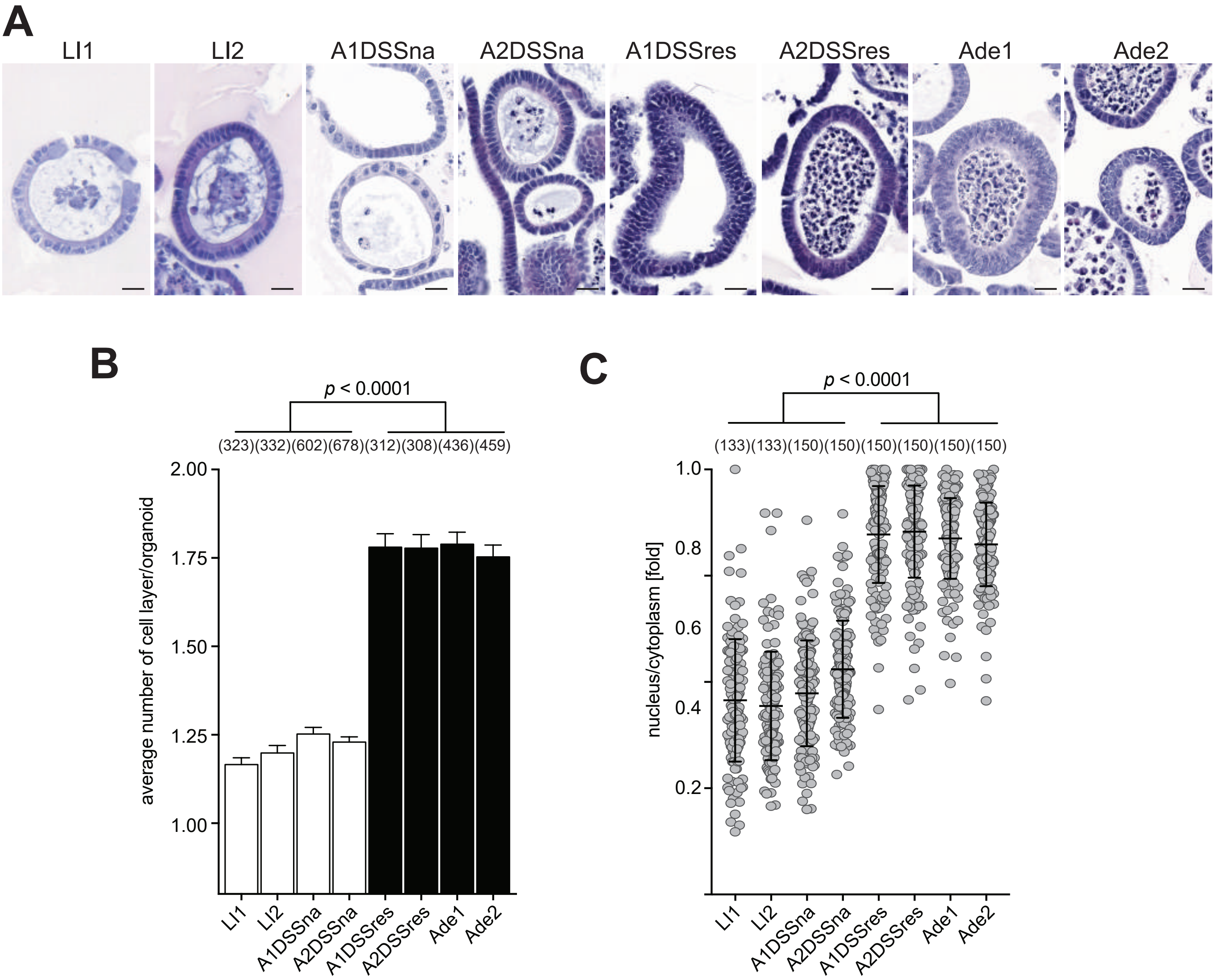

Figure S7. *In vivo* AOM treatment and *in vitro* DSS administration select for organoids phenotypically similar to adenoma organoids, related to Figure 4. (**A**) Representative H&E images of LI1, LI2, A1DSSna, A2DSSna, A1DSSres, A2DSSres, Ade1 and Ade2 organoids. Scale bar = 50 µm. (**B**) Quantification of single cell layer vs multiple cell layer organoid morpholo-gy (from **A**); numbers in parentheses represent number of analyzed organoids (mean±SEM), $p < 0.0001$. (**C**) Quantification of nuclear to cytoplasm ratio per organoid (from **A**); numbers in parentheses represent number of analyzed organoids (mean ±SD), $p < 0.0001$.

Figure S8

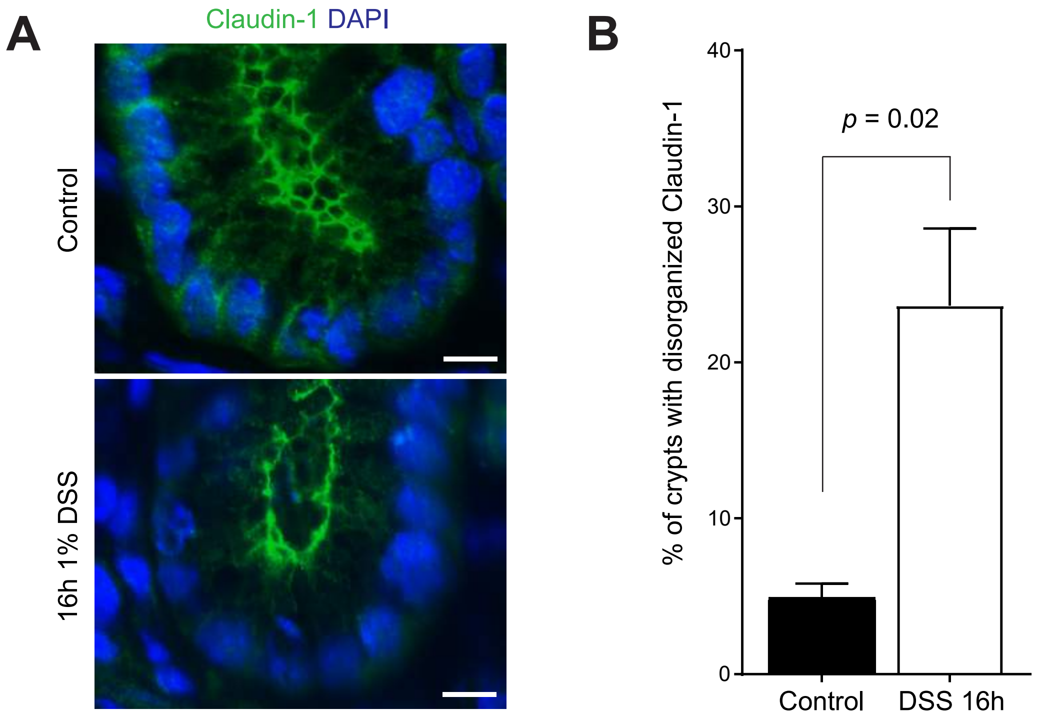

Figure S8. DSS treatment causes disorganization of the tight junction protein Claudin-1, related to Figure 7. (**A**) Distal colons from control mice and 1% DSS-treated mice (16h) were stained for Claudin-1. Scale bar = 10 μm. (**B**) shows quantification of the effect of DSS on disor-ganization of Claudin-1 from 3 independent repeats. p = 0.02, Student's t test.

Table S1

HGNC	Gene ID	Mouse Mutation	Human Equivalent	COSMIC	Organoid Line
Apc	11789	Q234*<br>Q471*<br>E1263K	Q236*<br>Q473*<br>E1265	1<br>n/a<br>n/a	A2DSS<br>A2DSS<br>A1DSS
Gsk3β	56637	P380S	P393	n/a	A1DSS
Axin2	12006	T686I	T689	n/a	A1DSS
Trp53	22059	A135T<br>V194M	A138T<br>V197M	2<br>6	A1DSS<br>A1DSS
Pik3ca	18706	G118D	G118D	18	A2DSS
Arid1a	93760	A2180V<br>P1575L	A2182fs<br>P1574L	1<br>1	A1DSS<br>A2DSS
Elf3	13710	S176L<br>D115N	P156<br>D96	n/a<br>n/a	A1DSS<br>A1DSS
Dnmt3a	13435	C516F	C520	n/a	A1DSS
Nf1	18015	A2V<br>V822I<br>V852M	A2<br>V820<br>V850	n/a<br>n/a<br>n/a	A1DSS<br>A1DSS<br>A1DSS
Kmt2b	75410	P586L	P577	n/a	A1DSS
Kmt2d	381022	P973L<br>P2232S<br>Q3600Afs<br>Q3601Hfs<br>Q4049del	P1008<br>P2275H<br>Q3602<br>Q3603<br>n/a	n/a<br>1<br>n/a<br>n/a<br>n/a	A2DSS<br>A1DSS<br>A1DSS<br>A1DSS<br>A2DSS
Casp8	12370	A399T	A414	n/a	A1DSS
Gata3	14462	T77I	T78	n/a	A1DSS
Crebbp	12914	V1372I	V1371F<br>V1371D	2<br>2	A1DSS<br>A1DSS
Cdk12	69131	T522I	T525	n/a	A2DSS
Mga	29808	S2373N	S2385	n/a	A2DSS
Tbx3	21386	E326D<br>E399D<br>A714T	D306<br>E381<br>A696E<br>A696V	n/a<br>n/a<br>1<br>1	A1DSS<br>A1DSS<br>A1DSS

Table S1. Mutations in colonic organoids derived from AOM-treated mice. 578 cancer-relevant mouse genes were screened by MSK-IMPACT. Mutations were identified in both DSS-naïve and DSS-resistant A1 and A2 organoid lines. Murine amino acid changes were mapped to their human counterparts and number of examples found in the COSMIC database are listed, if found (n/a=not available) (73). Gene ID numbers are those for mouse. Gene names from the HUGO Gene Nomenclature Committee (HGNC).
